# Enhanced machine learning—ensemble method for estimation of oil formation volume factor at reservoir conditions

**DOI:** 10.1038/s41598-023-42469-4

**Published:** 2023-09-14

**Authors:** Parsa Kharazi Esfahani, Kiana Peiro Ahmady Langeroudy, Mohammad Reza Khorsand Movaghar

**Affiliations:** 1 Department of Petroleum Engineering, Amirkabir University of Technology (Tehran Polytechnic), 424 Hafez Avenue, Box 15875-4413, Tehran, 1591634311 Iran; 2 Department of Mathematics and Computer Science, Amirkabir University of Technology (Tehran Polytechnic), 424 Hafez Avenue, Box 15875-4413, Tehran, 1591634311 Iran; 3 Department of Computer Engineering, Amirkabir University of Technology (Tehran Polytechnic), 424 Hafez Avenue, Box 15875-4413, Tehran, 1591634311 Iran

**Keywords:** Computer science, Chemical engineering

## Abstract

Since the oil formation volume factor (B_o_) is crucial for various calculations in petroleum engineering, such as estimating original oil in place, fluid flow in the porous reservoir medium, and production from wells, this parameter is predicted using conventional methods including experimental tests, correlations, Equations of State, and artificial intelligence models. As a substitute to conventional black oil methods, the compositional oil method has been recently used for accurately predicting the oil formation volume factor. Although oil composition is essential for estimating this parameter, it is time-consuming and cost-intensive to obtain through laboratory analysis. Therefore, the input parameter of dissolved gas in oil has been used as a representative of the amount of light components in oil, which is an effective factor in determining oil volume changes, along with other parameters, including pressure, API gravity, and reservoir temperature. This study created machine learning models utilizing Gradient Boosting Decision Tree (GBDT) techniques, which also incorporated Extreme Gradient Boosting (XGBoost), GradientBoosting, and CatBoost. A comparison of the results with recent correlations and machine learning methods adopting a compositional approach by implementing tree-based bagging methods: Extra Trees (ETs), Random Forest (RF), and Decision Trees (DTs), is then performed. Statistical and graphical indicators demonstrate that the XGBoost model outperforms the other models in estimating the B_o_ parameter across the reservoir pressure region (above and below bubble point pressure); the new method has significantly improved the accuracy of the compositional method, as the average absolute relative deviation is now only 0.2598%, which is four times lower than the previous (compositional approach) error rate. The findings of this study can be used for precise prediction of the volumetric properties of hydrocarbon reservoir fluids without the need for conducting routine laboratory analyses by only employing wellhead data.

## Introduction

Among the fluid properties of hydrocarbon reservoirs, the oil formation volume factor (B_o_) plays a vital role. This parameter indicates the change in the volume of produced oil from the reservoir to surface conditions. In fact, the volume of oil that enters the stock tank under surface conditions is less than the volume of oil produced in reservoir conditions that enter the production well. The oil volume change (from reservoir to surface conditions) is most affected by the significant pressure reduction below the bubble point and the resultant release of dissolved gases in oil, especially in large amounts of solution gases. Therefore, the oil formation volume factor defined as below is always equal to or greater than 1^1,2^.

1 $${B}_{o}= \frac{Reservoir \, oil \, volume \, at \, specified \, temperature \, and \, pressure}{Stock \, tank \, oil \, from \, reservoir \, oil}.$$

The unprecise prediction of this parameter could make various processes and calculations challenging for oil engineers. These processes and calculations include reservoir simulations, inflow performance, fluid flow in porous media, place-in-oil estimation, material balance, well test analysis, and economic analysis^3–17^

The ideal method to determine the PVT properties of oil samples is to use experimental tests, which are often costly and time-consuming. Hence, there have been numerous studies on predicting PVT properties using correlations^3,14,18–20^, equations^21^, and developing various artificial intelligence-based approaches^21–35^. Table 1 provides a brief overview of the advantages and disadvantages of the aforementioned methods. Despite the simplicity of applying correlations for predicting PVT properties, especially the B_o_ parameter, they produce significant errors, limiting their application in sensitive activities (for example, estimating original oil in place). Artificial Intelligence-based methods can adequately limit this error with time and cost savings.

**Table 1:** Advantages and disadvantages of previous employed methods.

Methods	Advantages	Disadvantages
Corrolations^3,14,18–20^	(1) *Simplicity* Correlations are often simple mathematical expressions that can be easily implemented and used without requiring complex calculations (2) *Quick calculations* Correlations are typically computationally efficient, allowing for fast calculations and analysis (3) *Data availability* Correlations are often developed based on large datasets and extensive experimental measurements, making them readily available for use	(1) *Limited accuracy* Correlations are empirical relationships derived from experimental data. As a result, they may not accurately capture the complex physics and fluid behavior of oil systems in all cases, leading to inaccuracies in predicting the B_o_ (2) *Applicability limitations* Correlations are usually developed for specific ranges of temperature, pressure, and fluid composition. Extrapolating their use beyond these ranges may lead to unreliable results (3) *Lack of customization* Correlations are general relationships that do not account for specific characteristics of a particular reservoir. They may not capture reservoir specific effects and variations, potentially leading to inaccuracies in B_o_ predictions
Equations of State^21^	(1) *Thermodynamic consistency* EOS provides a rigorous and thermodynamically consistent approach to model the behavior of fluids. They can handle a wide range of temperature, pressure, and fluid compositions, making them applicable to various reservoir conditions (2) *Accuracy* EOS can offer higher accuracy compared to correlations when properly calibrated and parameterized. They consider the intermolecular interactions and phase behavior of the fluid, providing more detailed predictions (3) *Customization* EOS can be customized and adjusted to match the specific characteristics of a particular reservoir. This allows for a more accurate representation of the fluid behavior and can improve B_o_ predictions	(1) *Complexity* EOS calculations are more complex and computationally demanding compared to correlations. They often require extensive fluid characterization and parameterization, which can be time-consuming and data-intensive (2) *Parameter uncertainty* Accurate parameter estimation and calibration are crucial for EOS models. Obtaining reliable and accurate parameters may require additional experimental data, and uncertainties in the parameters can impact the accuracy of the B_o_ predictions (3) *Implementation challenges* Proper implementation and usage of EOS models may require specialized software and expertise in fluid thermodynamics, which can pose challenges for some users
Nueral network^4,21–24,27,29,30,32^	(1) *Nonlinearity* Neural networks are capable of modeling nonlinear relationships in data, allowing them to capture complex patterns and make more accurate predictions (2) *Feature learning* Neural networks can automatically learn relevant features from raw data, reducing the need for manual feature engineering (3) *Adaptability* Neural networks can adapt and learn from new data, making them suitable for tasks where the underlying patterns or relationships change over time	(1) *Training complexity* Neural networks often require a large amount of training data and significant computational resources to train properly (2) *Black-box nature* Neural networks are often considered black-box models, making it challenging to interpret and understand the inner workings of the model (3) *Overfitting* Neural networks, especially with a large number of parameters, are prone to overfitting if not properly regularized or if the training data is limited or noisy (4) *Computational cost* Training and running neural networks can be computationally expensive, especially for deep architectures with numerous layers
Traditional machine learning^21,24,25,29,33,35^	(1) *Simplicity* Traditional machine learning algorithms are often simpler to understand and interpret compared to complex models like neural networks (2) *Interpretability* Traditional machine learning algorithms often provide transparent and interpretable models, allowing users to understand the factors driving the predictions (3) *Well-established theory* Traditional machine learning algorithms are based on well-established statistical and mathematical principles, allowing for a better understanding of their behavior	(1) *Limited capacity for complex patterns* Traditional machine learning algorithms may struggle to capture highly complex patterns in data, particularly those involving nonlinearity or high-dimensional relationships (2) *Feature engineering* Traditional machine learning algorithms often require manual feature engineering, where domain knowledge is needed to select relevant features and design appropriate representations (3) *Limited scalability* Some traditional machine learning algorithms may have limitations in handling large datasets or datasets with high-dimensional features (4) *Sensitivity to input data* Traditional machine learning algorithms may be sensitive to the quality and distribution of input data, which can affect their performance
Ensemble machine learning^21,29^	(1) *Improved accuracy* Ensemble methods combine multiple models, reducing bias and variance which leads to improved overall predictive accuracy (2) *Robustness* Ensemble methods can be more robust to outliers and noise in the data, as the combined predictions can mitigate the impact of individual model errors (3) *Generalization* Ensemble methods can capture a wider range of patterns and relationships in the data, enhancing their generalization capabilities (4) *Model diversity* Ensemble methods incorporate diverse models, leveraging different perspectives and reducing the risk of model limitations	(1) *Lack of transparency* Ensemble models can be less transparent and harder to interpret compared to single models, making it challenging to understand the underlying decision-making process

To estimate the B_o_ parameter with good accuracy, several studies have employed potent machine learning methods^21,24,25,29,33,35^ such as tree-based algorithms, Support Vector Machine (SVM), linear/non-linear regression, deep learning, and neural network^4,21–24,27,29,30,32^, and other network-based methods such as Adaptive Neuro-Fuzzy Inference System (ANFIS). These methods were developed based on experimental data from reservoirs in different regions, for this purpose, a part of the data is used for training and the other part is applied for testing the models. Moreover, several studies combined the aforementioned artificial intelligence methods with optimization algorithms^23,25–27,29,33,34^ such as Genetic Algorithm (GA), Simulated Annealing (SA), and Particle Swarm Optimizer (PSO) for optimizing input parameters. Utilizing an optimization algorithm prior to the development of a Machine Learning model confers benefits including improved performance, accelerated convergence, enhanced generalization, increased efficiency, customization according to specific requirements, and improved interpretability of the model. Also, it is worthy to be mentioned that, some studies have presented their results as correlations^14,23–27,30,31^. To better explain the development of the literature, some of the major studies in the past decade are discussed in the following.

Studies based on the development of correlations include Arabloo et al.^14^, who used LINGO, Fattah and Lashin^25^, who used the non-linear regression technique and Genetic Programming (GP) based on volatile oil reservoirs data bank, and Mahdiani and Norouzi^26^, who used the Simulated Annealing (SA) optimization method. The presented correlations for predicting B_o_ are based on common parameters such as reservoir temperature, solution oil–gas ratio, API gravity, and gas relative density. They all claimed that the proposed correlations improved the prediction accuracy compared to previous ones.

Saghafi et al.^27^ proposed models and correlations for predicting oil formation volume factor using Adaptive Neuro-Fuzzy Inference System (ANFIS). In addition to that, a functional correlation implementing the Genetic Programming (GP) model was proposed based on the aforementioned parameters.

In another study, Seyyedattar et al.^29^ used other tree-based methods such as Extra Tree (ET) in addition to ANFIS to estimate the oil formation volume factor. This study also extensively discussed the ET model’s remarkable capability to estimate the intended parameter with a wide range of features.

In another major study, Rashidi et al.^33^ combined Machine Learning with optimization methods to achieve improvement. This study employed two Machine Learning algorithms (Multi-layer Extreme Learning Machine (MELM) and Least Squares Support Vector Machine (LSSVM)) and two methods in order to optimize the parameters (a Genetic Algorithm (GA) and a Particle Swarm Optimizer (PSO)). It is also noteworthy that applying the PSO method instead of GA halved the prediction error.

All of the reviewed studies that used artificial intelligence to predict B_o_ were based on the black oil method and conventional features (such as reservoir temperature, solution gas-oil ratio, API gravity, and gas relative density).

Larestani et al.^21^ utilized multiple machine learning techniques such as ETs, RF, DTs, generalized regression neural networks, and cascade-forward backpropagation network in conjunction with radial basis function and multilayer perceptron neural networks to estimate oil formation volume factor based on the compositional oil method. This study used oil composition (obtained from oil composition analysis) and other common input parameters to introduce ETs as the superior model based on statistical and graphical comparisons. To express this model’s efficiency, various comparisons were made with correlations, previous machine learning methods, and Equations of State (EOS).

Aforementioned studies used machine learning and neural network to estimate the B_o_ parameter. Despite their efficiency, all these methods were effectively Black Boxes that hid the exact relationship between inputs and outputs and prevented distinguishing these functions clearly. To overcome this limitation, Wood and Choubineh^28^ used the Transparent Open Box (TOB) learning network algorithm that led to more logical and accurate predictions. Note that the proposed method was only evaluated for predicting the oil formation volume factor in the bubble point.

In all of these studies, the key issue addressed is the more precise estimation of B_o_ with reduced computational errors. Furthermore, it is essential that these methods are optimized in terms of both time and computational costs. To achieve this, innovative artificial intelligence-based techniques have been employed, along with their simultaneous integration.

This study aimed to accurately estimate the oil formation volume factor (B_o_) using machine learning methods in various reservoir pressure and temperature ranges through black oil parameters and without implementing the results of oil composition analysis. The database used for training and testing the models covers a wide range of PVT data from Iran’s oil reservoirs, including 1241 data points from Constant Composition Expansion (CCE), Differential Liberation (DL), and separator tests. Three advanced soft computing approaches that rely on Gradient Boosting Decision Tree (GBDT) were utilized. These include XGBoost, GradientBoosting, and CatBoost. Hence, the developed models can reliably predict B_o_ in other Iranian oil reservoirs.

In this study, the reservoir pressure parameter is also used as an effective parameter along with other input parameters, including reservoir temperature, API gravity, and the solution gas-oil ratio (R_s_) of the samples. To express the performance of the GBDT-developed models, quantitative and qualitative analyzes as well as comparison with previous Machine Learning approaches, including Random Forest (RF), Decision Trees (DTs), and Extra Trees (ETs) based on the oil composition method, is used. The advantage of the proposed method is the non-dependence of B_o_ estimation on its values at lower pressures (e.g., bubble point pressure).

The remaining part of the document is structured as follows: The “Model” section provides an overview of the fundamental principles and algorithms of each soft computing technique that has been implemented. The section titled “Results and discussion” outlines the approach taken, model creation, and provides an analysis of the findings and subsequent discussions. Finally, the “Conclusion” section summarizes the key findings of the study.

## Model

The study utilizes an emerging Machine Learning technique known as ensemble, which combines multiple classifiers to enhance the robustness and improve the accuracy of classification performance. This technique is more effective in dealing with noise compared to single-classifier methods^36,37^. This research employs three ensemble techniques that utilize a Gradient Boosting Decision Tree algorithm: GradientBoosting, CatBoost, and XGBoost^38–40^. Some reasons for implementing boosting methods can be discussed as follows:

1. *Parallelization and scalability* Many boosting implementations, such as XGBoost are designed to be highly parallelizable and scalable. They can efficiently utilize parallel computing resources, such as multi-core CPUs or distributed computing frameworks, to speed up the training process and handle large-scale datasets.
2. *Improved predictive accuracy* Boosting methods excel at improving predictive accuracy compared to other traditional machine learning algorithms. They combine multiple weak models (often decision trees) to create a strong ensemble model that can capture complex relationships in the data. By iteratively focusing on the samples that are difficult to predict, boosting methods gradually improve the overall accuracy of the model.
3. *Robustness to overfitting* Boosting methods are effective in reducing overfitting. They utilize techniques such as regularization to mitigate the risk of overfitting the training data. This allows boosting models to generalize well to unseen data and perform consistently on different datasets.

In order to facilitate a more comprehensible perception, Table 2 provides a concise overview of the advantages, disadvantages, and applications of each utilized model.

**Table 2:** Advantages, disadvantages and applications of each utilized model.

	Gradient Boosting^43^	CatBoost^46^	XGBoost^48^
Advantages	(1) Recommendation systems (2) Natural language processing (3) Image and video analysis (4) Fraud detection	(1) Built-in handling of categorical features (2) Automatic handling of missing values (3) Excellent handling of large datasets	(1) High predictive performance (2) Efficient implementation (3) Regularization techniques to prevent overfitting (4) Feature importance ranking
Disadvantages	(1) Sensitive to hyperparameter tuning (2) Prone to overfitting with complex datasets (3) Lack of built-in handling for categorical features	(1) Longer training time for large datasets (2) Relatively high memory consumption (3) Requires more computational resources	(1) Requires tuning of hyperparameters 2) Limited handling of categorical features (3) Difficult to interpret complex models
Applications	(1) Predictive modeling in various domains (2) Financial risk analysis (3) Healthcare and medical research (4) Customer churn prediction	(1) Recommendation systems (2) Natural language processing (3) Image and video analysis (4) Fraud detection	(1) Classification and regression problems (2) Feature selection and ranking (3) Anomaly detection (4) Time series forecasting

### GradientBoosting^41,42^

The boosting technique is focused on iterating and reevaluating errors at each step to create a robust learner by combining multiple weaker learners. The training data used for the model can be defined as $x=\{{x}_{1},{x}_{2}, \dots , {x}_{n}\}$ representing the features of interest and y as the target data. In essence, this method aims to find the approximate value of $\widetilde{F}\left(x\right)$ for *F(x)* based on the following condition:

2 $$\widetilde{F}\left(x\right)=\mathrm{arg}\underset{F\left(x\right)}{\mathrm{min}}{L}_{y,x}\left(y,F\left(x\right)\right),$$

where, ${L}_{y,x}\left(y,F\left(x\right)\right)$ is the cost function and $\mathrm{arg}\underset{F\left(x\right)}{\mathrm{min}}{L}_{y,x}\left(y,F\left(x\right)\right)$ is the value of *F(x)* for which ${L}_{y,x}\left(y,F\left(x\right)\right)$ achieves its minimum. The cost function enhances the accuracy of parameter prediction by attaining the minimum value. Each weak learner endeavors to improve upon and reduce the errors of the previous weak learner. Ultimately, the objective is to obtain the desired regression tree function (i.e., $h({x}_{i};a)$) where parameter *a* represents a weak learner. Each decision tree is then adjusted and aligned to its determined slope. ${F}_{m}\left(x\right)$ is updated in the final step based on the iterations performed^43^. For more detailed information, please refer to the Supplementary File—Sect. 2.1—GradientBoosting.

### CatBoost^44,45^

CatBoost is a relatively new Gradient Boosting Decision Tree (GBDT) method. GBDT is known to perform well when applied to datasets containing numerical features. However, some datasets may contain string features such as gender or country names. These features may greatly impact the accuracy of our final predictions, so it is crucial not to ignore or eliminate them. Therefore, it is customary to convert categorical (string) features into numerical features before training a dataset.

Unlike some other GBDT-based methods, CatBoost offers a notable advantage by being able to handle categorical features during the training process. As mentioned earlier, categorical features are inherently non-numerical. To incorporate them into our model, we need to convert them into numerical features before commencing the training process. For detailed information about the conversion methods and how CatBoost addresses potential issues Prokhorenkova et al.^46^ that may arise during this process, please refer to the Supplementary File—Sect. 2.2—CatBoost.

### XGBoost^47^

The Extreme Gradient Boosting (XGBoost) algorithm, which was developed and introduced by Chen et al.^48^, belongs to modern Machine Learning techniques based on Gradient Boosting Decision Trees. This algorithm aims to minimize errors and maximize adaptability by creating a large number of trees (e.g., *k*) to approximate the estimated value as closely as possible. By combining weak learners, the algorithm builds a strong learner. However, in this algorithm, weak learners are constructed through residual fitting^49,50^. The XGBoost model extends the cost function by incorporating first-order Taylor information and presenting second-order derivative information. This enhancement enables faster convergence during the learning process. Additionally, the XGBoost algorithm includes a regularization component in the cost function, which helps control complexity and reduces the risk of overfitting. For a more detailed understanding of the general process of the XGBoost algorithm, please refer to the Supplementary File—Sect. 2.3—XGBoost.

**Figure 1:**
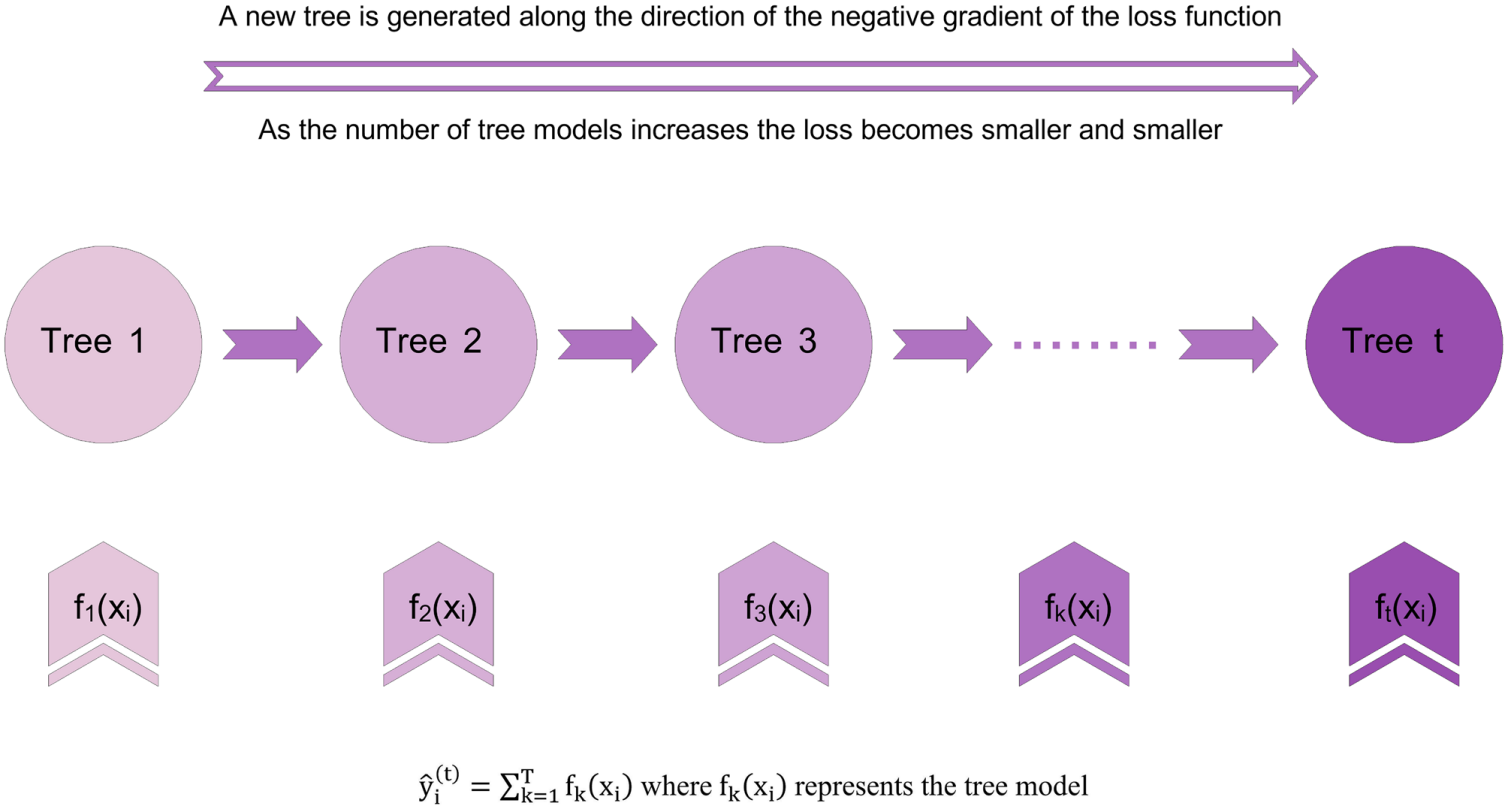
Schematic of XGBoost algorithm.

To provide a more tangible comprehension, Fig. 1 illustrates the proposed algorithm structure^51^.

The name and version of the packages used in the analysis and model development are as follows:

- **NumPy**: 1.22.4;
- **pandas**: 1.5.3;
- **scikit-learn**: 1.2.2;
- **catboost**: 1.2;
- **xgboost**: 1.7.6;
- **seaborn**: 0.12.2;
- **matplotlib**: 3.7.1.

## Results and discussion

### Model development

The databank is obtained from a series of PVT tests on various samples of Iranian oil in a wide pressure range above and below each sample’s bubble point. At pressures exceeding the bubble point, the B_o_ parameter is obtained from DL and separator tests. At the same time, it is necessary to use CCE and separator tests to determine these parameters at pressures below the bubble point. The following correlations are used to obtain this parameter from the results of mentioned experiments^1^:

3 $${B}_{o}= {\left(\frac{{V}_{t}}{{V}_{b}}\right)}_{CCE}{B}_{oSb}\, \left(P \ge {P}_{b}\right),$$

4 $${B}_{o}= {B}_{oD} \frac{{B}_{oSb}}{{B}_{oDb}}\, \left(P < {P}_{b}\right).$$

${\left(\frac{{V}_{t}}{{V}_{b}}\right)}_{CCE}$ is the total relative volume by the CCE test. ${B}_{oSb}$ is the oil formation volume factor at bubble point pressure obtained from the separator test. ${B}_{oDb}$ is the oil formation volume factor at bubble point pressure from the DL test and ${B}_{oD}$ is the oil formation volume factor from the DL test at the desired pressure.

Therefore, a total of 1241 experimental data points, which adequately represent Iranian crude oil samples, were collected and used to develop efficient models for B_o_ estimation with greater accuracy. The features used in each sample include reservoir pressure and temperature, API gravity, and solution gas-oil ratio (R_s_) which has physical base and are also implemented in known correlations that are used in B_o_ estimation.

**Figure 2:**
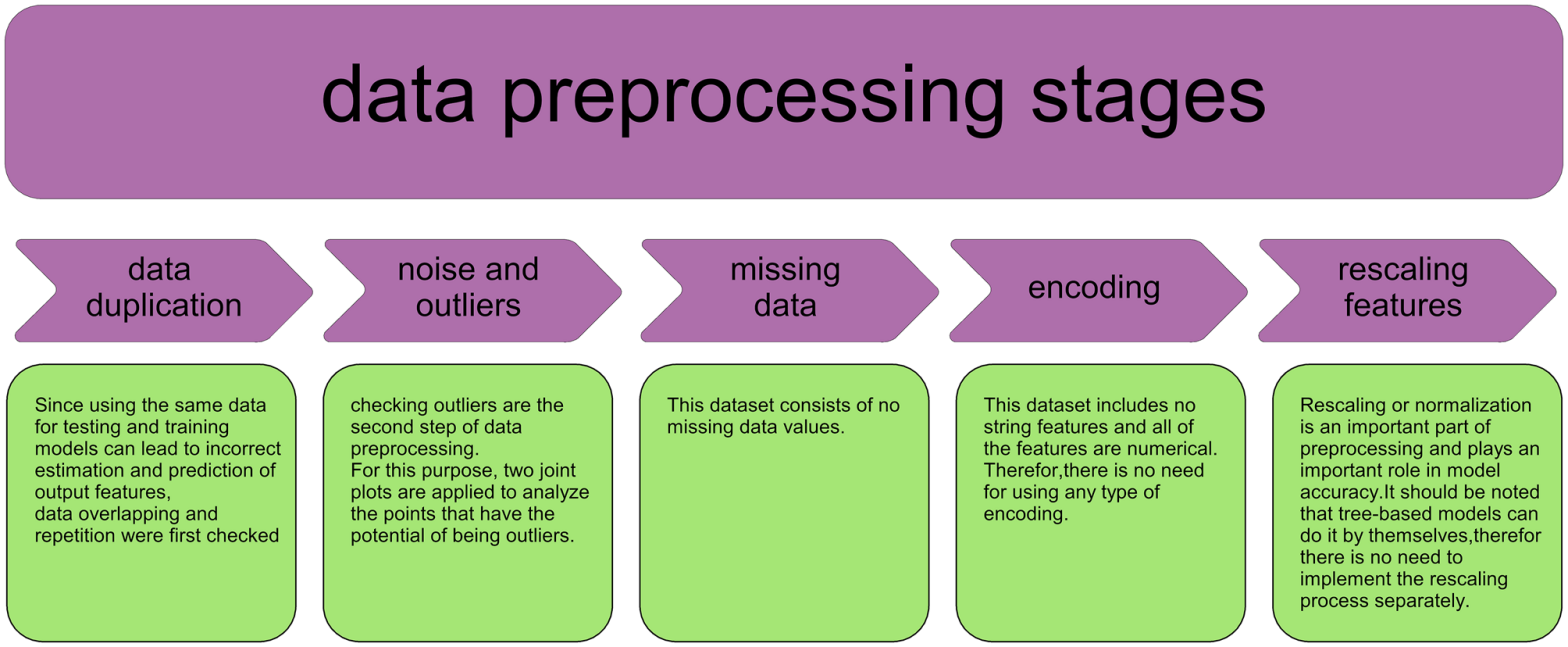
Data preprocessing steps.

It is important to note that the methodology employed in this approach relies on black oil, which reduces the number of features to save time and reduce memory consumption and can lead to more efficient commercial simulators. Also, five data preprocessing stages are applied which are summarized in Fig. 2*.* Running preprocessing stages before model development offers advantages such as improved data quality, enhanced feature representation, and better handling of missing or irrelevant data, leading to improved model performance and generalization capabilities. Additionally, preprocessing allows efficient data transformation, normalization, and scaling, enabling the model to effectively learn patterns and relationships in the data.

**Figure 3:**
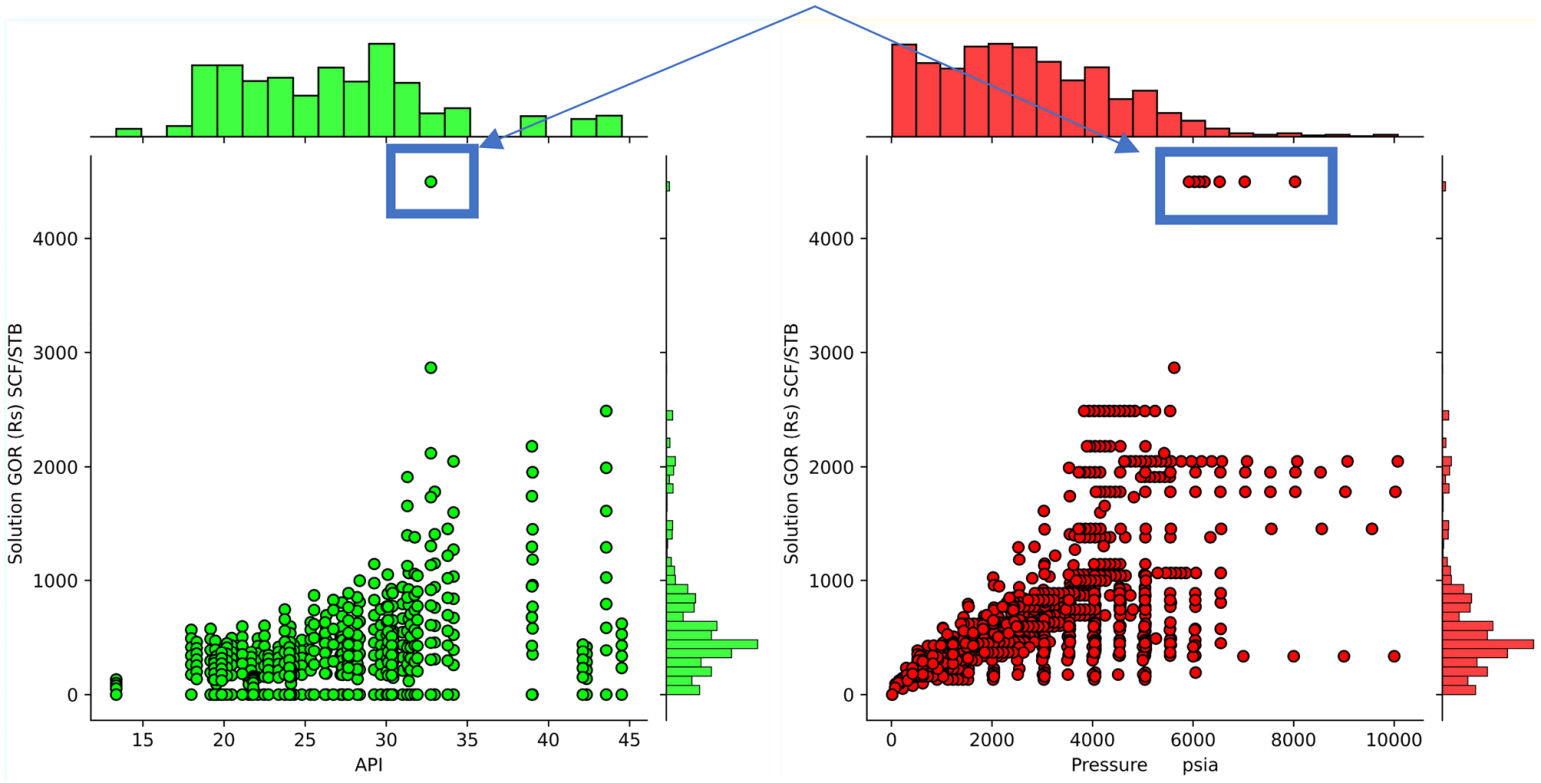
Data joint plots for outliers’ detection.

By analyzing the results presented in Fig. 3 and taking into account expert opinion, 8 outliers were detected among the collected data. Although these points are valid, they exhibit a significant departure from the majority of the data samples. Their considerable deviation from the mean strongly influences the parameters and coefficients estimated by Machine Learning models, which may compromise predictive performance. Hence, these data points are excluded from the training and testing datasets.

Table 3 provides a comprehensive overview of whole data (including the train and test data ranges) utilized for constructing the models.

**Table 3:** Statistical ranges and parameters related to inputs/outputs employed for developing models.

No	Parameters	Unit	Count	Mean	Std	Min	25%	50%	75%	Max
1	Pressure	psi	1233	2618.08	1757.94	14.70	1230.00	2428.00	3833.00	10,072.00
2	Temperature	°F	1233	215.43	40.21	110.00	190.00	208.00	247.00	290.00
3	Solution GOR (R_s_)	SCF/STB	1233	607.46	496.64	0.00	327.77	473.52	771.18	2866.89
4	API	–	1233	26.90	6.49	13.35	21.67	26.74	30.39	44.52
5	Oil formation volume factor (B_o_)	bbl/STB	1233	1.42	0.30	1.02	1.25	1.33	1.50	2.83

To ensure robust and dependable results, it is important to note that the databank was randomly split into two subsets. The first subset, comprising 80% of the data, was used to train the models, while the second subset, which contained the remaining 20% of the data, was used to evaluate the effectiveness of the models. Therefore, the reliability of developed models can be compared to blind cases.

Regarding the evaluation of the model using testing data, a comprehensive analysis has been conducted to ensure that the testing data falls within the range of the training data. This evaluation was based on the information provided in the Tables 4 and 5. Statistical variables, including mean and standard deviation, were calculated for the training data. Subsequently, it was verified that the values reported for the testing data aligns within an acceptable range determined by these statistical measurements. It is hereby confirmed that the testing data demonstrates a strong alignment with the characteristics and distributions observed in the training data. Consequently, this validation ensures the performance of the model and its generalizability to real-world scenarios.

**Table 4:** Statistical ranges for training data.

No	Parameters	Unit	Count	Mean	Std	Min	25%	50%	75%	Max
1	Pressure	psi	987	2585.46	1765.57	14.70	1223.00	2423.00	3833.00	10,072.00
2	Temperature	°F	987	214.90	40.29	110.00	190.00	208.00	247.00	290.00
3	Solution GOR (R_s_)	SCF/STB	987	605.00	502.94	0.00	314.85	473.52	771.18	2866.89
4	API	–	987	26.96	6.51	13.35	21.67	26.74	30.39	44.52
5	Oil formation volume factor (B_o_)	bbl/STB	987	1.42	0.30	1.02	1.25	1.33	1.50	2.83

**Table 5:** Statistical ranges for testing data.

No	Parameters	Unit	Count	Mean	Std	Min	25%	50%	75%	Max
1	Pressure	psi	246	2748.43	217.55	14.70	1535.00	2525.00	3837.00	8535.00
2	Temperature	°F	246	217.55	39.89	110.00	190.00	208.00	248.00	290.00
3	Solution GOR (R_s_)	SCF/STB	246	617.00	471.49	0.00	340.91	478.83	755.57	2487.61
4	API	–	246	26.67	6.42	13.35	21.52	26.30	30.26	44.52
5	Oil formation volume factor (B_o_)	bbl/STB	246	1.43	0.29	1.03	1.26	1.34	1.48	2.52

Grid Search is a hyperparameter tuning technique used in machine learning to find the optimal values for a set of hyperparameters that can produce the best model performance. Hyperparameters are model parameters that cannot be learned from the data and should be specified beforehand. Grid Search involves defining a set of values for each hyperparameter, creating a grid of all possible combinations of hyperparameter values, and then evaluating each combination using a performance metric such as accuracy or mean squared error. The combination of hyperparameter values that produces the best performance on the evaluation metric is then selected as the optimal hyperparameter^52^.

Table 6 displays the control parameters for algorithms utilized in this paper, which are the outcome of hyperparameters.

**Table 6:** Control parameters employed in development and application of soft computing techniques.

	Parameters	Value
GradientBoosting	n-Estimators	120
	Max depth	5
	Learning rate	0.10
	Subsample	1
	Alpha	0.90
	Min samples split	2
XGBoost	n-Estimators	94
	Max depth	9
	Learning rate	0.08
	Subsample	0.75
	Gamma	0
	Col sample by tree	1
CatBoost	Depth	7
	Learning rate	0.07
	Iterations	300
	Best model min trees	1
	Bootstrap type	MVS
	Leaf estimation method	Newton

### Performance evaluation

This study employed various statistical and graphical comparisons to examine the capability and adequacy of the models. The correlations for obtaining the statistical indicators are presented in the following:

1. Average absolute relative deviation (AARD):
  5 $$AARD\%=\frac{1}{N}\sum_{i=1}^{N}\left|\frac{{O}_{iexp}-{O}_{ipred}}{{O}_{iexp}}\right|\times 100.$$
2. Coefficient of determination (R^2^):
  6 $${R}^{2}=1-\frac{{\sum }_{i=1}^{N}{\left({O}_{iexp}-{O}_{ipred}\right)}^{2}}{{\sum }_{i=1}^{N}{\left({O}_{ipred}-\overline{O }\right)}^{2}}.$$
3. Root mean square error (RMSE):
  7 $$RMSE=\sqrt{\frac{1}{N}\sum_{i=1}^{N}{\left({O}_{iexp}-{O}_{ipred}\right)}^{2}}.$$

In Eqs. (5), (6), and (7) ${O}_{i}$ represents the output (oil formation volume factor (B_o_)), and exp represents the actual B_o_ values, while pred represents the estimated B_o_ values. Furthermore, $\overline{\mathrm{O} }$ denotes the mean of the outputs, and N represents the total number of data points.

To demonstrate the strength of our models, we conducted a tenfold cross-validation on the training dataset. The process of cross-validation involves dividing the training set into k subsets, training a model with k − 1 folds, and validating the model with the remaining data. The performance of the model is then evaluated as the average of the values obtained for each fold. In this study, a tenfold cross-validation was performed, and the resulting RMSE-score was found to be 0.0198 for XGBoost as shown in Table 7. It could be seen later that this reported RMSE-score suggests that the XGBoost model performed well not only on the 20% of data used for test but also on the dataset used for training, indicating that the model is highly accurate and reliable.

**Table 7:** Performance measure reported by tenfold cross-validation.

Folds	XGBoost (RMSE)	GradientBoosting (RMSE)	CatBoost (RMSE)
Fold-1	0.0189	0.0236	0.0241
Fold-2	0.0207	0.0280	0.0289
Fold-3	0.0251	0.0225	0.0224
Fold-4	0.0145	0.0172	0.0196
Fold-5	0.0181	0.0259	0.0286
Fold-6	0.0176	0.0232	0.0209
Fold-7	0.0259	0.0183	0.0187
Fold-8	0.0193	0.0212	0.0203
Fold-9	0.0156	0.0288	0.0270
Fold-10	0.0224	0.0240	0.0255
Folds mean	0.0198	0.0232	0.0236

**Figure 4:**
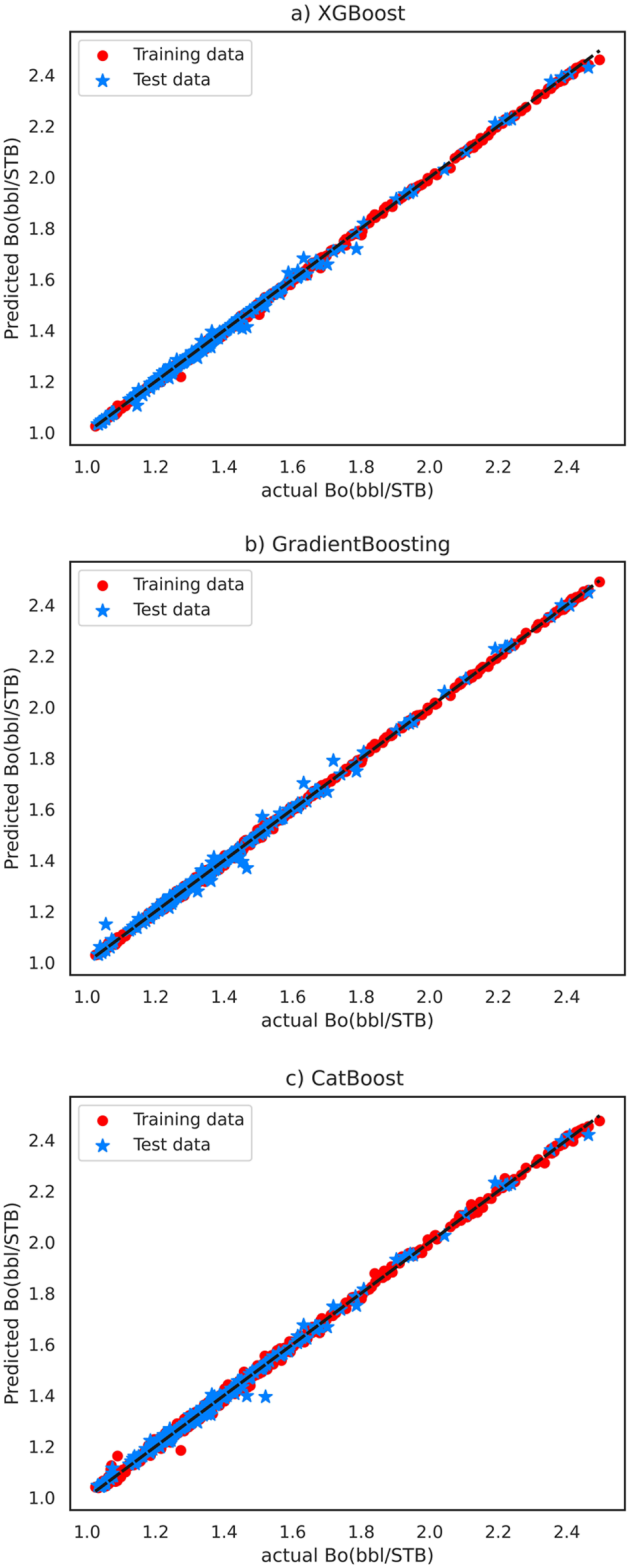
Cross plots of the implemented models: (**a**) XGBoost, (**b**) GradientBoosting, and (**c**) CatBoost.

Combining graphical evaluation with statistical indicators facilitates the examination of models in terms of accurate B_o_ estimation. In Fig. 4*.* According to cross plots the uniform distribution of predictions along the X–Y axis suggests that these models produce accurate predictions. The majority of the test data, with minimal deviation from the X–Y axis in the case of XGBoost, indicates excellent performance and suggests that XGBoost outperforms other methods in terms of efficiency. The overlap between the predicted values and the actual values in the cross-plot evaluation method can be used to assess how accurately and effectively the models perform.

Table 8 reports the statistical indicators of the developed models. The results illustrate that with average absolute relative deviation (AARD) and coefficient of determination (R^2^) of 0.2598% and 0.9994, respectively, the XGBoost model outperforms the other models for B_o_ estimation. In the following, these statistical indicators are used for comparing the models with reviewed methods in terms of performance.

**Table 8:** Statistical indices used for describing the performance of proposed models.

Models	Train			Test			Overall		
	RMSE	R^2^	AARD (%)	RMSE	R^2^	AARD (%)	RMSE	R^2^	AARD (%)
XGBoost	0.0046	0.9997	0.2085	0.0111	0.9980	0.4646	0.0059	0.9994	0.2598
GradientBoosting	0.0057	0.9996	0.2948	0.0159	0.9960	0.6110	0.0078	0.9989	0.3581
CatBoost	0.0105	0.9987	0.5293	0.0154	0.9962	0.9603	0.0114	0.9982	0.5615

The time and memory occupied by each model are additional performance indicators that can be used alongside error analysis based on statistical indicators. Therefore, the average Training Time, Inference time, and occupied memory are reported in Table 9 which indicates that XGBoost is significantly faster while requiring less Time and Memory for train and test.

**Table 9:** Time and memory assessment of each modelling approach.

Models	Training time (s)	Inference time (s)	Memory (MB)
XGBoost	0.11	0.001	271
GradientBoosting	0.21	0.002	281
CatBoost	0.68	0.004	254

The study finds that XGBoost has improved upon the Gradient Boosting Decision Tree (GBDT) technique in several key areas. Firstly, XGBoost utilizes a second-order Taylor expansion with both first and second orders as improved residuals, whereas traditional GBDT only uses the first-order Taylor expansion. This feature allows XGBoost to capture more complex relationships between features and enhance its prediction power. Secondly, XGBoost incorporates a regularization term in its objective function to control the model’s complexity and prevent overfitting. This regularization improves the model’s generalization performance on new data. Overall, the study concludes that the combination of these features makes XGBoost a highly effective and versatile Machine Learning method. Lastly, XGBoost uses the random forest column sampling method to further reduce the chance of overfitting. Hence, the XGBoost model has shown excellent learning performance and training speed^45^.

**Figure 5:**
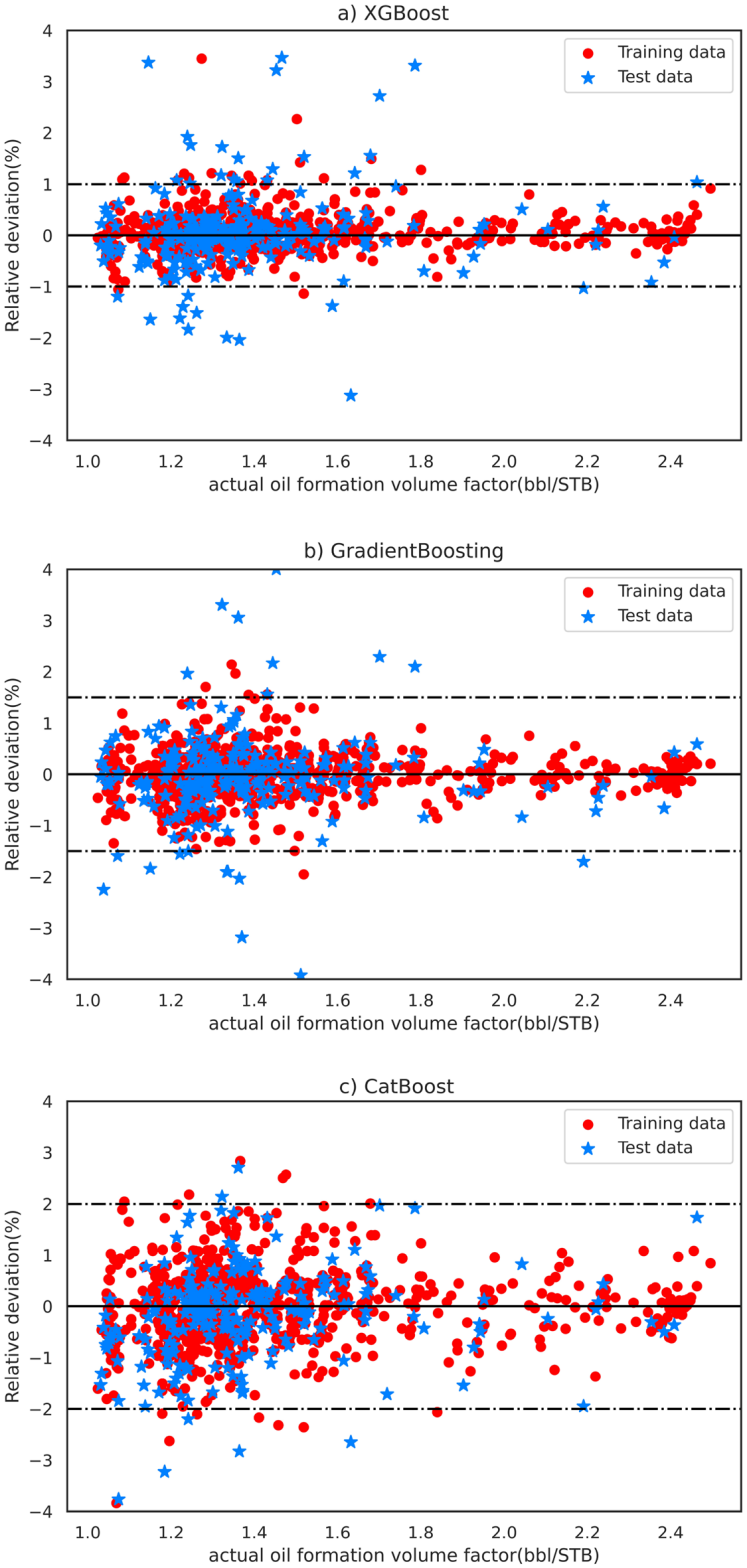
Relative deviation (%) of estimated oil formation volume factor (B_o_) values using the (**a**) XGBoost, (**b**) GradientBoosting, and (**c**) CatBoost model for test and train data points.

An alternative approach for evaluating model performance entails analyzing the predictive deviation of each model with respect to the B_o_ value acquired from experimental tests across the entire dataset. In this assessment, narrower ranges of deviation signify superior performance in parameter prediction and estimation. Figure 5 shows the relative deviation of the developed models, revealing that the XGBoost model achieves less than 1% absolute relative deviation for the majority of the dataset. This outcome serves as evidence of the accuracy and efficiency of the XGBoost model.

### Comparison of the developed models with previous approaches

The previous sections employed statistical indicators and graphical tools to show the B_o_ estimation performance of the developed models for various pressure ranges. The XGBoost model, a machine learning method discussed in this study, had better performance among the others. Larestani et al.^21^ presented a Machine Learning approach based on the bagging method using compositional oil features^21^. This method was shown to be superior to previous Machine Learning methods and various Equations of State using the statistical indicators presented in the Supplementary File—Sect. 3—Comparison with the preexisting approaches. Therefore, the results of this study will only be compared to Larestani et al.^21^ results in the following. Also note that for a fair comparison, the same databank was used for testing and training processes in the present study and Larestani et al.^21^.

#### Comparison with compositional study

Using the oil composition method, 18 features in the normal method, including oil composition (methane to C_11_ and non-hydrocarbons), specific gravity and molecular weight of C_12_^+^, reservoir temperature and pressure, and 7 features by division of oil components into three subgroups in the lumped method, Larestani et al.^21^ estimated B_o_ as the only desirable output parameter. As mentioned before, the features used for developing the models in this study include API gravity, temperature, pressure, and R_s_. Table 10 compares the models from this study with the top three models of Larestani et al.^21^, which include tree-based bagging methods. As shown in Table 10, Larestani et al.^21^ introduced the Extra Trees model in lumped mode as the optimal model. Analysis of indicators suggests that all the models, especially the XGBoost technique, outperform the methods proposed by Larestani et al.^21^. As a novel and advanced Machine Learning model, XGBoost has reduced the ETs model’s error (Larestani et al.^21^ superior model) down to a quarter despite using fewer features (4 compared to 18 in normal mode/4 compared to 7 in lumped mode). This was achieved while presenting more accurate estimations and time and cost savings (independent of oil composition analysis), suggesting that it can be practical and economical for simulations.

**Table 10:** Performance of the developed models in comparison with the compositional models.

Models	Train			Test			Overall		
	RMSE	R^2^	AARD (%)	RMSE	R^2^	AARD (%)	RMSE	R^2^	AARD (%)
XGBoost^a^	0.0046	0.9997	0.2085	0.0111	0.9980	0.4646	0.0059	0.9994	0.2598
GradientBoosting^a^	0.0057	0.9996	0.2948	0.0159	0.9960	0.6110	0.0078	0.9989	0.3581
CatBoost^a^	0.0105	0.9987	0.5293	0.0154	0.9962	0.9603	0.0114	0.9982	0.5615
Normal Random Forest^b^	0.0425	0.9866	0.9390	0.0541	0.9745	1.0424	0.0451	0.9844	0.9597
Normal Decision Trees^b^	0.0645	0.9703	1.2312	0.0430	0.9797	1.4002	0.0608	0.9717	1.2650
Normal Extra Trees^b^	0.0261	0.9944	1.2132	0.0342	0.9929	1.3511	0.0279	0.9940	1.2408
Lumped Random Forest^b^	0.0395	0.9894	0.9600	0.0250	0.9898	1.0426	0.0370	0.9895	0.9766
Lumped Decision Trees^b^	0.0966	0.9293	1.3422	0.0510	0.9793	1.4343	0.0893	0.9389	1.3607
Lumped Extra Trees^b^	0.0248	0.9954	1.1404	0.0320	0.9915	1.2785	0.0264	0.9947	1.1681

^a^This study.

^b^Larestani et al.^21^.

**Figure 6:**
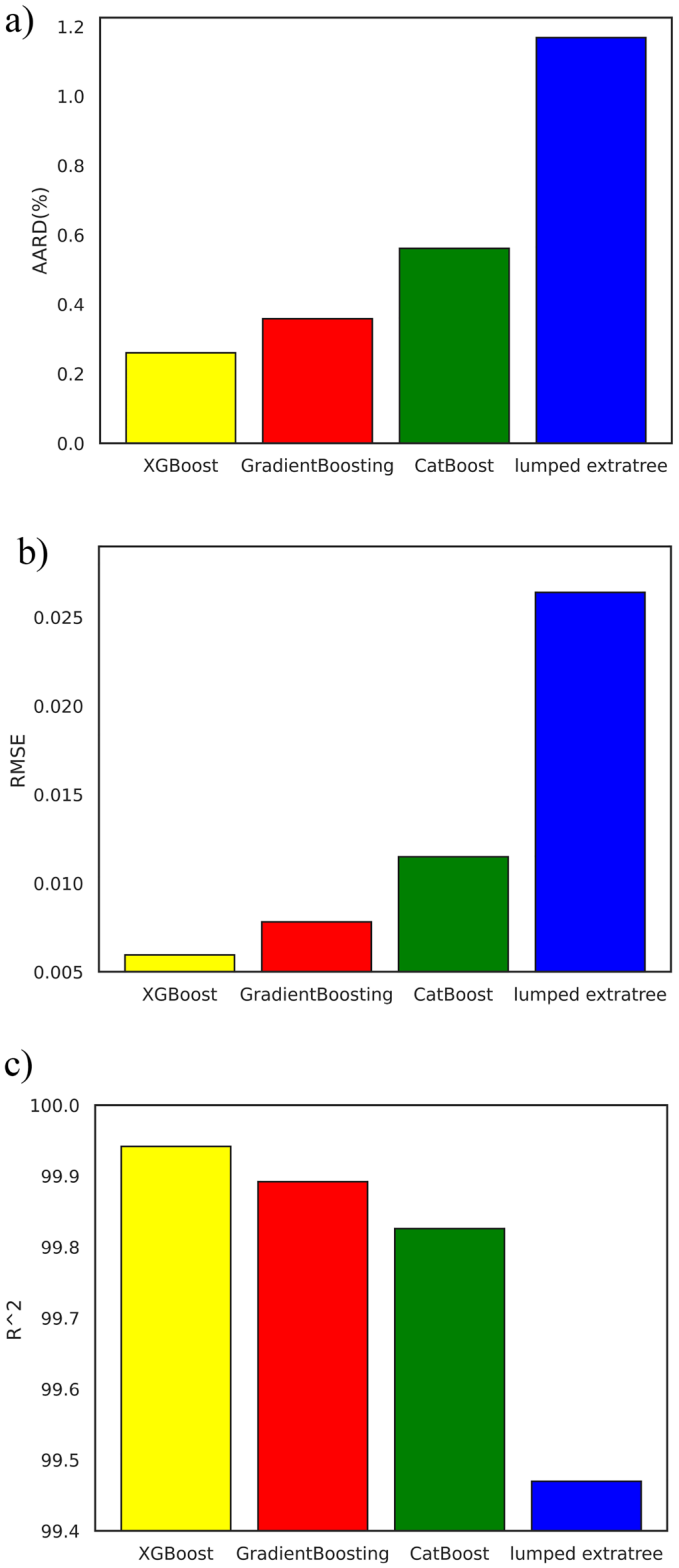
Error bar charts of the developed models in this study in comparison with the best compositional model (ETs) of Larestani et al.^21^ based on (**a**) AARD (%), (**b**) RMSE, and (**c**) R^2^ in estimating oil formation volume factor (B_o_).

This paper provided train errors besides test errors to indicate whether the model suffers from overfitting or not. If the model has a significantly lower train error than the test error, it indicates potential overfitting. This difference suggests that the model is fitting the training data very well but struggles to generalize to new, unseen data. In this regard Table 10 and Fig. 6 compares the bar charts of AARD (%), RMSE, and R^2^ statistics for these models and the superior model from Larestani et al.^21^. Figure 6 shows a clear graphical representation of the superior performance of the developed models, especially XGBoost, in all error measurement statistics.

As mentioned in the “Model” section, the black oil model was selected in order to reduce the number of input features as a consequence for time and memory savings. It should be noted that, likewise, Larestani et al.^21^ selected the lumped models to significantly reduce the number of features. To compare runtimes and occupied memory for the XGBoost method used in this study and the lumped models in Larestani et al.^21^ study, the average runtimes and occupied memory are reported in Table 11. The results demonstrate that XGBoost requires significantly less computing time and memory. It is worthy to be noted that for having a fair comparison in computation time, all the developed models within this study, as well as developed models in Larestani et al.^21^ study are compiled with CPU.

**Table 11:** Time and memory assessment comparison of the XGBoost model vs. Larestani et al.^21^ lumped models.

Models	Training time (s)	Inference time (s)	Memory (MB)
XGBoost	0.11	0.001	271
DTs	0.28	Not reported	313
RF	0.62	Not reported	339
ETs	0.99	Not reported	295

**Figure 7:**
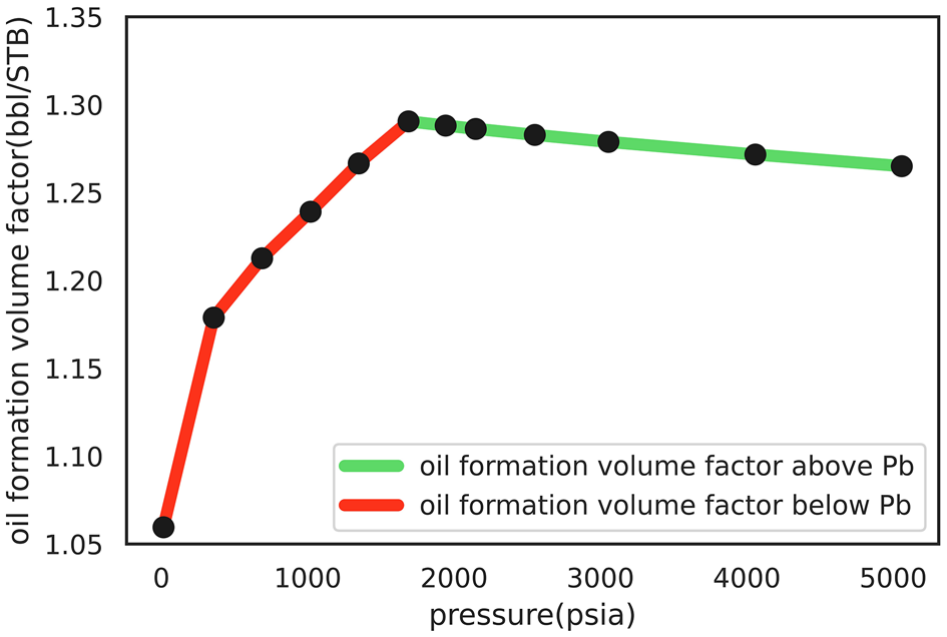
Oil formation volume factor (B_o_) vs. pressure curve.

**Figure 8:**
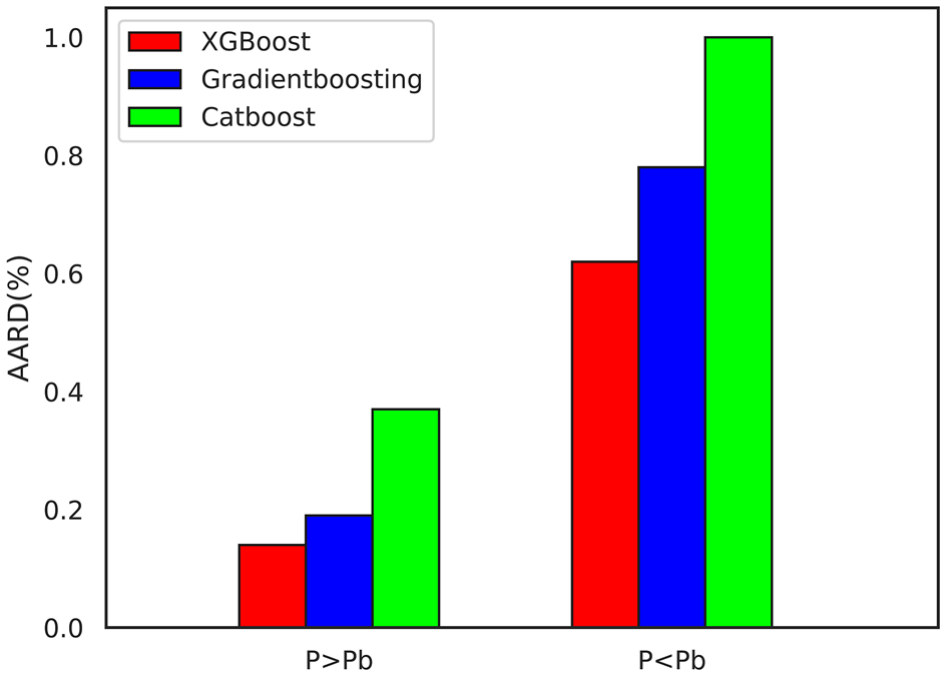
Accuracy of developed models in predicting oil formation volume factor (B_o_) for two different pressure ranges (above and below bubble point pressure).

Figure 7 shows changes in B_o_ for different pressures relative to the bubble point suggesting that B_o_ changes were lower for pressures exceeding the bubble point. These limited changes could be attributed to the stable oil composition in pressures exceeding the bubble point where B_o_ changes are merely due to oil expansion in the reservoir. Meanwhile, solution gases are lower at pressures below the bubble point, which reduces oil volume closer to surface conditions. In fact, changes to oil composition due to solution gas evolved affect B_o_ at pressures lower than the bubble point, and the reduction in solution gases reduces B_o_. Therefore, as a representative of oil composition^53^ and a crucial parameter of oil volume change, R_s_ can be included in the features set. To better illustrate the greater efficiency of the developed models, Fig. 8 shows the bar chart of their prediction error in the two pressure ranges (higher and lower than the bubble point).

In order to establish a reliable basis for comparison, this study provides a comprehensive analysis of the reasons behind the superiority of boosting methods over other bagging methods. Specifically, while the Extra Tree algorithm employs bagging, the XGBoost, CatBoost, and GradientBoosting algorithms utilize boosting techniques. Both boosting and bagging are ensemble methods that aim to improve the accuracy of Machine Learning models. However, their approaches differ, and which method is better depends on the specific problem and dataset.

Bagging, or bootstrap aggregating, is a method where multiple models are trained on different subsamples of the data with replacement, and the final prediction is a combination of the predictions from all the models. Bagging can reduce variance and overfitting.

On the other hand, Boosting is an iterative method that trains multiple weak models sequentially, where each subsequent model tries to correct the errors of the previous one. Boosting aims to reduce bias and improve model performance.

Several studies have compared the performance of boosting and bagging on various datasets, and the results are mixed. Some studies have shown that boosting outperforms bagging, while others have shown the opposite. A review of ensemble methods by Buciluǎ et al.^54^ found that boosting and bagging have similar performance on many datasets, but boosting tends to perform better on datasets with a small number of features.

In our study, there are 4 features and it’s obvious that 4 features aren’t high for Machine Learning tasks so boosting methods can perform better than bagging methods.

### Samples

**Figure 9:**
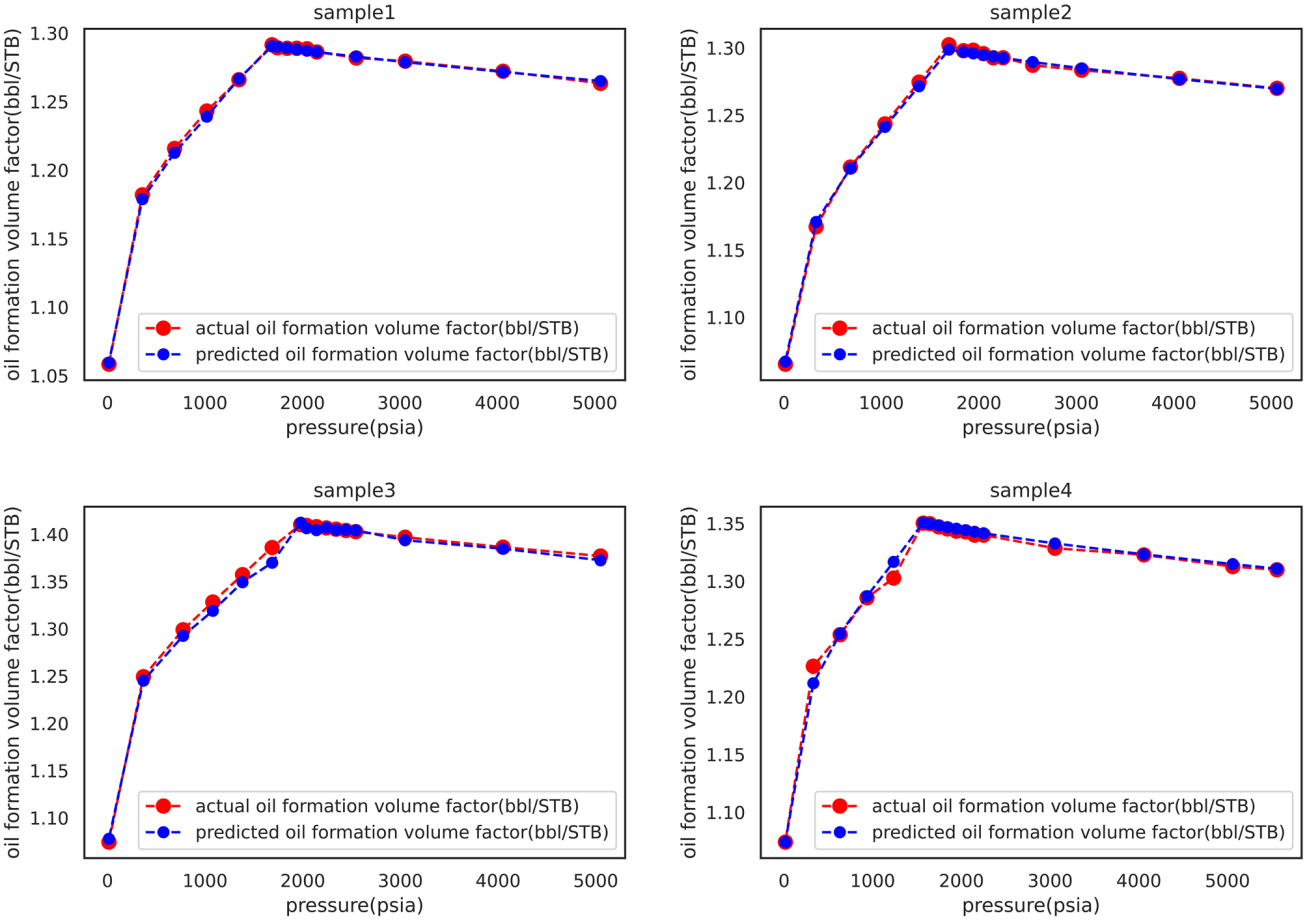
Graphical illustration for comparison between experimental B_o_ values and the XGBoost model estimations of four Iranian oil samples at different pressures.

Table 12 presents the experimental B_o_ values and the XGBoost model estimations for four Iranian oil samples at different pressures. Also, in order to provide a better outlook a graphical illustration is presented corresponding to each sample in Fig. 9. The figure evidently demonstrates the capability of the proposed model in reproducing the physical trend at different pressures which is in agreement with the general knowledge. Hence, it can be concluded more confidently that the XGBoost model can accurately estimate B_o_ regardless of the pressure range and oil type.

**Table 12:** Experimental B_o_ values and the XGBoost model estimations for four Iranian oil samples at different pressures.

Sample 1			Sample 2		
P (psia)	Real B_o_ (bbl/STB)	Model B_o_ (bbl/STB)	P (psia)	Real B_o_ (bbl/STB)	Model B_o_ (bbl/STB)
5058	1.264993	1.263273	5058	1.269586	1.269956
4057	1.271601	1.272116	4057	1.276778	1.277404
3054	1.278784	1.279456	3054	1.284824	1.283406
2551	1.282781	1.281855	2551	1.289471	1.287139
2148	1.286200	1.286202	2248	1.292532	1.292635
2046	1.287120	1.288550	2148	1.293607	1.292635
1942	1.288076	1.288924	2046	1.294728	1.295759
1842	1.289012	1.288924	1942	1.295899	1.298283
1743	1.289957	1.289285	1842	1.297055	1.297930
**1688.769**	**1.290442**	**1.291468**	**1692.429**	**1.298694**	**1.302322**
1348	1.266570	1.265986	1386	1.271466	1.274578
1019	1.239040	1.243145	1036	1.241328	1.243556
688	1.212583	1.215799	682	1.210465	1.211540
358	1.178781	1.182006	330	1.170797	1.167173
14.7	1.059500	1.058335	14.7	1.067100	1.065139

Sample 3			Sample 4		
P (psia)	Real B_o_ (bbl/STB)	Model B_o_ (bbl/STB)	P (psia)	Real B_o_ (bbl/STB)	Model B_o_ (bbl/STB)
5049	1.372511	1.376968	5557	1.310795	1.309808
4050	1.384720	1.386455	5058	1.314791	1.312617
3047	1.393742	1.396921	4057	1.323210	1.322753
2543	1.404223	1.402851	3054	1.332665	1.328542
2442	1.404594	1.404133	2248	1.341454	1.339894
2341	1.404594	1.405437	2148	1.342729	1.339894
2241	1.406945	1.406750	2046	1.344060	1.342788
2140	1.404457	1.408102	1942	1.345451	1.343368
2039	1.406495	1.409479	1842	1.346824	1.345261
**1977.934**	**1.412340**	**1.410265**	1743	1.348221	1.347062
1686	1.369929	1.386076	1643	1.349671	1.349845
1383	1.349317	1.357261	**1568.62**	**1.350668**	**1.350203**
1079	1.319025	1.328358	1236	1.316720	1.302710
774	1.292498	1.299005	935	1.287005	1.285658
368	1.245166	1.249311	632	1.254527	1.253684
14.7	1.077700	1.074200	330	1.211657	1.226408
			14.7	1.074100	1.074109

Significant values are in bold.

## Conclusion

The AARD errors associated with the machine learning algorithms based on GBDT, namely XGBoost, GradientBoosting, and CatBoost, in the present study are reported 0.2598%, 0.3581%, and 0.5615% respectively. Hence, the XGBoost model has attained the best results. On the other hand, the results from previous study concerning the utilization of bagging models demonstrate that the lumped Extra Tree model (the best-reported approach by Larestani et al.^21^, exhibits the AARD error rate of 1.1681%. As a result, the XGBoost model has successfully improved the error value by 0.9% in comparison with lumped ETs.

The most significant advantage of the current study, is considering only four input parameters without the need of applying oil composition data compared to the bagging models implementing compositional approach along with a higher number of input parameters (18 parameters for the normal case/7 parameters for lumped case).

Additionally, another advantage is the development of a single model for all pressure regions in the reservoir, ranging from very low pressures to pressures exceeding the bubble point. Despite of this study, previous studies have employed two separate models for higher and lower pressure regions of the bubble point.

Furthermore, the favorable performance of XGBoost can be attributed to the following factors:

1. To elaborate on the XGBoost algorithm, it is a relatively new method based on GBDT that creates trees of equal depths consecutively, making it faster than other GBDT-based models due to parallel processing. It also employs L1 and L2 regularization techniques to mitigate overfitting.
2. L1 regularization encourages parameters to approach zero, effectively removing the impact of certain features, while L2 regularization reduces the magnitude of weights without forcing them to become precisely zero.
3. The XGBoost model exhibits the capability to handle missing or NaN (Not a Number) data values, enhancing its robustness and practicality in real-world applications.

In addition, the universal application of the developed models is predicting volumetric properties of newly discovered reservoirs using limited wellhead and reservoir data, without the need for running routine PVT laboratory tests. These models can be trained using available fluid samples from pre-developed fields in a specific region of the world and then utilized for other fields in the same region.

One of the limitations of the conducted study is the utilization of certain hyperparameters with default values, which can be optimized in future studies using appropriate optimization methods.

### Supplementary Information

Supplementary Information.

## Data Availability

The data will be available upon request. The corresponding author (MRK) should be contacted for this purpose.

## Abbreviations

AARD: Average absolute relative deviation, %
AI: Artificial intelligence
ANFIS: Adaptive neuro-fuzzy inference system
bbl: Barrel
CCE: Constant composition expansion
CPU: Central processing unit
DL: Differential liberation
DTs: Decision Trees
EOS: Equations of State
ETs: Extra Trees
GA: Genetic Algorithm
GBDT: Gradient Boosting Decision Tree
GOR: Gas-oil ratio, SCF/STB
GP: Genetic programming
LSSVM: Least squares support vector machine
MELM: Multi-layer extreme learning machine
ML: Machine learning
NaN: Not a number
NN: Artificial neural network
OFVF: Oil formation volume factor, bbl/STB
PSO: Particle Swarm Optimizer
PVT: Pressure–volume–temprature
RF: Random Forest
RMSE: Root mean square error, unit of the original value
SA: Simulated annealing
SCF: Standard cubic foot
Std: Standard deviation, unit of the original value
STB: Standard barrel
SVM: Support Vector Machine
TOB: Transparent Open Box
XGBoost: Extreme Gradient Boosting

## Parameters

a: Representative of a weak learner
$B_{o}$: Oil formation volume factor, bbl/STB
$B_{ob}$: Oil formation volume factor at bubble point pressure, bbl/STB
$B_{oD}$: Oil formation volume factor from DL test at desired pressure, bbl/STB
$B_{oDb}$: Oil formation volume factor at bubble point pressure from DL test, bbl/STB
$B_{oSb}$: Oil formation volume factor at bubble point pressure obtained from separator test
F(x): Objective function
R_s_: Solution gas-oil ratio, SCF/STB
$h\left( {x_{i} ;a} \right)$: Desired regression tree function
k: Number of subsets
L1: Overfitting preventer regularization
L2: Overfitting preventer regularization
$$L_{y,x} \left( {y,F\left( x \right)} \right)$$: Cost function
N: Number of data points
$O_{iexp}$: Experimental/actual output
$O_{ipred}$: Predicted/estimated output
$\overline{O}$: Mean of outputs
P: Pressure, psia
P_b_: Bubble point pressure, psia
R^2^: Coefficient of determination
$\left( {\frac{{V_{t} }}{{V_{b} }}} \right)_{CCE}$: Total relative volume by CCE test
x: Features of interest
y: Target data
