# Supplementary material for: Enhanced machine learning—ensemble method for estimation of oil formation volume factor at reservoir conditions
Source: Sci Rep. 2023 Sep 14;13:15199. doi: 10.1038/s41598-023-42469-4 (PMC10502101; doi:10.1038/s41598-023-42469-4)
Supplement: Supplementary file 1 — Supplementary Information. [file 41598_2023_42469_MOESM1_ESM.docx]

**Enhanced machine learning - ensemble method for estimation of oil formation volume factor at reservoir conditions**

Parsa Kharazi Esfahani ^1,2^, Kiana Peiro Ahmady Langeroudy ^1,3^, [Mohammad Reza Khorsand Movaghar](https://www.researchgate.net/profile/Mohammad-Reza-Khorsand-Movaghar-2) ^1, *^

^1^ Department of Petroleum Engineering, Amirkabir University of Technology (Tehran Polytechnic), Box 15875-4413, 424 Hafez Avenue, Tehran, 1591634311, Iran

^2^ Department of Mathematics and computer science, Amirkabir University of Technology (Tehran Polytechnic), Box 15875-4413, 424 Hafez Avenue, Tehran, 1591634311, Iran

^3^ Department of Computer Engineering, Amirkabir University of Technology (Tehran Polytechnic), Box 15875-4413, 424 Hafez Avenue, Tehran, 1591634311, Iran

^*^  Corresponding author: Email: [m.khorsand@aut.ac.ir](mailto:m.khorsand@aut.ac.ir), Tel.: +98 21 64545133

1^st^ author email: [pkharazi1080@aut.ac.ir](mailto:pkharazi1080@aut.ac.ir), 2^nd^ author email: [kiana.peiro80@aut.ac.ir](mailto:kiana.peiro80@aut.ac.ir)

**Supplementary File**

# **1. Ensemble Classifier**

An ensemble classifier is a method that uses or combines multiple classifiers to improve robustness as well as to achieve an improved classification performance from any of the constituent classifiers. Furthermore, this technique is more resilient to noise compared to the use of a single classifier. This method uses a divide and conquers approach where a complex problem is decomposed into multiple sub-problems that are easier to understand and solve. Ensemble approaches [[1](#_ENREF_1),[2](#_ENREF_2)] have the advantage that they can be made to adapt to any changes in the monitored data stream more accurately than single model techniques. An ensemble classifier has better accuracy than single classification techniques. The success of the ensemble approach depends on the diversity in the individual classifiers with respect to misclassified instances [[3](#_ENREF_3)]. According to [Polikar [4]](#_ENREF_4), there are four ways to achieve this diversity, the first is to use different training data to train single classifiers, the second is to use different training parameters, the third is to use different features to train the classifiers and the final one is to combine different types of the classifier. [Dietterich [5]](#_ENREF_5) reported that there are three main reasons why an ensemble classifier is usually significantly better than a single classifier. Firstly, the training data does not always provide sufficient information for selecting a single accurate hypothesis. Secondly, the learning processes of the weak classifier might be imperfect, and thirdly, the hypothesis space being searched might not contain the true target function while an ensemble classifier can provide a good approximation.

It is well known in the data mining literature that the appropriate combination of several weak classifiers can yield a highly accurate global classifier [[6](#_ENREF_6)]. Hence, three different ensemble classifier techniques, called bagging, boosting, and stacking, using various weak classifiers, such as the nearest neighbor, decision tree, rule induction, and naïve Bayes [[2](#_ENREF_2),[7](#_ENREF_7),[8](#_ENREF_8)]; are introduced here.

## **1.1. Bagging**

Bagging, which means bootstrap aggregation, is one of the simplest but most successful ensemble methods for improving unstable classification problems. For example, weak classifiers, such as decision tree algorithms, can be unstable, especially when the position of a training point changes slightly and can lead to a very different tree. This method is usually applied to decision tree algorithms, but it also can be used with other classification algorithms such as naïve Bayes, nearest neighbor rule induction, etc. The bagging technique is very useful for large and high-dimensional data, such as intrusion datasets [[2](#_ENREF_2)], where finding a good model or classifier that can work in one step is impossible because of the complexity and scale of the problem. Bagging was first introduced by [Breiman [9]](#_ENREF_9) to reduce the variance of a predictor. It uses multiple versions of a training set which is generated by a random draw with the replacement of N examples where N is the size of the original training set. Each of these data sets is used to train a different model. The outputs of the models are combined by voting to create a single output. Details of the bagging algorithm and its pseudo-code were given in [[10](#_ENREF_10)].

• Random Forest: The random forest classifier consists of a combination of tree classifiers where each classifier is generated using a random vector sampled independently from the input vector, and each tree casts a unit vote for the most popular class to classify an input vector [[11](#_ENREF_11)].

• Extra Tree: The Extra-Trees algorithm builds an ensemble of the unpruned decision or regression trees according to the classical top-down procedure [[12](#_ENREF_12)].

## **1.2. Boosting**

Boosting, which was introduced by [Bartlett, et al. [13]](#_ENREF_13), is an ensemble method for boosting the performance of a set of weak classifiers into a strong classifier. This technique can be viewed as a model averaging method and it was originally designed for classification, but it can also be applied to regression. Boosting provides sequential learning of the predictors. The first one learns from the whole data set, while the following learns from training sets based on the performance of the previous one. The misclassified examples are marked and their weights are increased so they will have a higher probability of appearing in the training set of the next predictor. It results in different machines being specialized in predicting different areas of the dataset [[5](#_ENREF_5),[14](#_ENREF_14)].

[Dietterich [5]](#_ENREF_5) established that boosting is more accurate than bagging.

Some boosting methods are AdaBoost, CatBoost, GBM, and XGBoost.

• GBM: The gradient boosting algorithm fits weak learners to loss function and each weak learner model aims to correct errors made by previous weak learner models. This can strengthen the prediction performance and reduce the prediction error of the model.

• Adaboost: AdaBoost algorithm is one of the most widely used boosting techniques for constructing a strong classifier as a linear combination of weak classifiers [[2](#_ENREF_2)].

• Catboost: CatBoost is a new gradient boosting decision tree (GBDT) algorithm that can handle categorical features well.

• XGBoost: The extreme gradient boosting algorithm (XGBoost) is a supervised gradient boosting-based ensemble learning algorithm proposed by [Chen, et al. [15]](#_ENREF_15). This algorithm aims to create a K regression tree to obtain the predicted value of the tree group as close to the true value as possible and achieve the greatest generalization ability.

## **1.3. Stacking**

Stacking or stacked generalization is a different technique of combining multiple classifiers. Unlike bagging and boosting, stacking is usually used to combine various classifiers, e.g. decision tree, neural network, rule induction, naïve Bayes, logistic regression, etc. Stacking consists of two levels which are base learner as level-0 and stacking model learner as level-1. Base learner (level-0) uses many different models to learn from a dataset. The outputs of each of the models are collected to create a new dataset. In the new dataset, each instance is related to the real value that it is supposed to predict. Then that dataset is used by the stacking model learner (level-1) to provide the final output [[14](#_ENREF_14)]. For example, the predicted classifications from the three base classifiers, naïve baye Bayes cision tree, and rule induction can be used as input variables into the nearest neighbor classifier as a stacking model learner, which will attempt to learn from the data how to combine the predictions from the different models to achieve the best classification accuracy [[10](#_ENREF_10)].





Supplementary Figure S1. examples of various machine learning approaches and algorithms [[16](#_ENREF_16)].

# **2. Model**

In the present study, the ensemble type of machine learning method, an emerging line of research, is employed. An ensemble classifier integrates multiple classifiers to increase robustness and represent an improved version of classification performance from any of the constituent classifiers. Additionally, this technique, in comparison to a single classifier technique, is more resilient to noise [[17](#_ENREF_17)]. The following ensemble methods are used in this study: GradientBoosting, CatBoost, and XGBoost machines that all these methods are developed using a gradient boosting decision tree [[18](#_ENREF_18),[19](#_ENREF_19)].

**2.1. GradientBoosting** [[20](#_ENREF_20)]

The boosting technique focuses on iteration and reconsideration of the errors in each step to develop a strong learner by integrating multiple weak learners. The data selected to train the model can be defined as below by assuming $x=\{x_{1},x_{2}, \ldots, x_{n}\}$ as the features of interest and y as the target data: $\left\{ \left( x_{i}, y_{i} \right) \right\} for i = 1, 2, \ldots, n with x_{i}\in R^{n} and y_{i}\in R$ .In general, this method aims to find the approximate value of $\tilde{F}\left( x \right)$for *F(x)* according to this conditions:

$\tilde{F}\left( x \right)=\arg\min_{F\left( x \right)} L_{y,x}\left( y,F\left( x \right) \right) (1)$

Where, $L_{y,x}\left( y,F\left( x \right) \right)$ is the cost function and $\arg\min_{F\left( x \right)} L_{y,x}\left( y,F\left( x \right) \right)$is the value of *F(x)* for which $L_{y,x}\left( y,F\left( x \right) \right)$achieves its minimum. The cost function improves the parameter prediction accuracy by reaching the smallest value. It is defined as the squared error: $L\left( y,F\left( x \right) \right)=\left( y-F\left( x \right) \right)^{2}$ In any case, there will always be an unavoidable error when an objective function (i.e., F(x)) is being estimated. This error can be either low or high based on the efficiency of the developed model.

Each of the weak learners tries to improve and reduce the previous weak learner’s error. The GradientBoosting algorithm adapts weak learners to the cost function as much as possible in order to increase the model accuracy and decrease errors. In the first step of the GradientBoosting algorithm, a base learner is defined as $F_{0}\left( x \right)$, which is often considered a fixed function for simplicity. In the next step, this algorithm benefits from gradient descent to minimize the predefined cost function. The gradient descent takes proportionate steps with negative slopes in the cost function to make the result reach the local minimum.

The cost function slope is obtained from **Equation (2)**:

$$\tilde{y}_{i}=-\left[ \frac{\partial L\left( y_{i},F\left( x_{i} \right) \right)}{\partial F\left( x_{i} \right)} \right]_{F_{\left( x \right)}=F_{m-1}\left( x \right)}, i=1,2,\ldots, n. (2)$$

If the regression tree function (i.e.,$h(x_{i};a)$) is used for parameter *a* representing a weak learner, the slope calculation range can be developed and generalized. In this function, $x_{i}$is defined as the input parameter, whereas *a* is defined as the parameter that should be determined [[21](#_ENREF_21)].

The desired tree is obtained by solving **Equation (3)**:

$$a_{m}=\arg\min_{a,\beta} \sum_{i=1}^{n} \left[ \tilde{y}_{i}-\beta h\left( x_{i};a \right) \right]^{2} (3)$$

Where *β* denotes the weighted coefficient (expansion of each weak learner’s coefficients) and $a_{m}$ is the parameter obtained from the *m*th iteration, respectively. Each decision tree is matched and adapted to its determined slope. After $\rho_{m}$(the optimal length) is determined, $F_{m}\left( x \right)$ is updated in the final step based on each iteration. The GradientBoosting algorithm is formalized by Algorithm 1 [[21](#_ENREF_21)].

Supplementary Algorithm S1: GradientBoosting

*1.* $F_{0}\left( x \right)=\arg\min_{\rho} \sum_{i=1}^{n} L\left( y_{i} , \rho\right)$

*2. For m = 1 to M do;*

*3.* $\tilde{y}_{i}=-\left[ \frac{\partial L\left( y_{i},F\left( x_{i} \right) \right)}{\partial F\left( x_{i} \right)} \right]_{F_{\left( x \right)}=F_{m-1}\left( x \right)} , i=1,\ldots, n$

*4.* $a_{m}=\arg\min_{a,\beta}\sum_{i=1}^{n} \left[ \tilde{y}_{i}-\beta h\left( x_{i};a \right) \right]^{2}$

*5.* $\rho_{m}=\arg\min_{\rho} \sum_{i=1}^{n} L\left( y_{i}-F_{m-1}\left( x_{i} \right)+\rho h\left( x_{i};a_{m} \right) \right)$

*6.*$F_{m}\left( x \right)=F_{m-1}\left( x \right)+\rho_{m}h\left( x ;a_{m} \right)$

*7. End for*

*End algorithm*

**2.2. CatBoost** [[22](#_ENREF_22),[23](#_ENREF_23)]

CatBoost is a relatively novel GBDT based method that demonstrated satisfactory results in Kaggle competitions. It also yielded considerable precisions in those competitions. A feature of GBDT is that it operates properly on datasets with numerical features. However, some datasets may include string features (e.g., gender or country) rather than merely numerical features. Hence, these features might be of great importance and have substantial effects on the accuracy of our final prediction, it is impossible to ignore or remove them. Therefore, it is customary to convert categorical (string) features into numerical features before a dataset is trained. Unlike some other GBDT based methods, an outstanding advantage of the CatBoost model is that it can handle categorical features in the training process.

As defined earlier, categorical features are non-numerical. So, for using them in our model, we must first convert them into numbers and then begin the training process of the model. One-Hot-Encoding is one of the methods for converting categorical features into numerical ones. This technique is applicable in both preprocessing and training. However, encoding in training data points will yield far better results. It is essential to note that CatBoost utilizes this method for optimal performance.

There are other ways to handle categorical features. Another method is presented below:

First, a dataset is constructed as **Equation (4)**:

$D=\left\{ \left( X_{i},Y_{i} \right) \right\}_{i=1,\ldots,n} \left\{ \begin{aligned} Xi =\left( x_{i, 1},\ldots, x_{i, m} \right) m is the number of features \\ Y_{i}\in R value of the target \end{aligned} \right. (4)$

_n is the number of data points_

To eliminate a categorical feature, we substitute it with the average target value derived from the entire training data.

Accordingly, **Equation (5)** is used instead of $x_{i,k}$.

$\frac{\sum_{j=1}^{n} \left[ x_{j,k}=x_{i\cdot k} \right]{.Y}_{j}}{\sum_{j=1}^{n} \left[ x_{j,k}=x_{i\cdot k} \right]} \left[ x_{j,k}=x_{i\cdot k} \right]= \left\{ \begin{aligned} 1 if x_{j,k}=x_{i\cdot k} \\ 0 if x_{j,k}\neq x_{i\cdot k} \end{aligned} \right. (5)$

where [·] denotes Iverson brackets

One of the problems with this solution is that it may overfit the model. To solve this problem, an alternative solution was proposed. For this purpose, the generated dataset (i.e., D) is divided into two sections: 1. training and 2. calculating the statistics.

Although this method handles the problem of overfitting to some extent, it reduces the quantity of data for training the model. Evidently, the availability of more data points can lead to better training in the model. In fact, the model can observe and analyze various data in this case.

CatBoost is designed to reduce the overfitting of the model. Unlike the aforementioned solution, it does not divide the data and uses the entire data to train the model. In other words, we apply a random permutation of the dataset and then we calculate the mean of the target value for each example with the same category value placed before the given one in the permutation.

Consider that $\sigma=\left( \sigma_{1},\ldots,\sigma_{n} \right)$ is the permutation, we substitute $x_{\sigma_{p},k}$ with

$\frac{\sum_{j=1}^{p_{-1}} \left[ x_{\sigma_{j,k}}=x_{\sigma_{p,k}} \right]\cdot y_{\sigma j}+\beta\cdot p}{\sum_{j=1}^{p_{-1}} \left[ x_{\sigma_{j,k}}=x_{\sigma_{p\cdot k}} \right]+\beta} (6)$

P: prior value $\beta$: weight of the prior

It is worthy to be noted that, using the prior value diminishes the effect of data noise.

Another challenge that arises while converting categorical features into numerical ones would be the possible change in the primary distribution of the features. The problem is that it causes deviation in the solution. CatBoost offers a solution, which is described ahead.

Theoretical analysis to overcome the gradient bias called ordered boosting, was proposed by [Prokhorenkova, et al. [24]](#_ENREF_24). The pseudo-code of ordered boosting is expressed in Algorithm 2:

Supplementary Algorithm S2: Ordered boosting

*Input:* $\left\{ \left( X_{k}, Y_{k} \right) \right\}_{k=1}^{n}$ *ordered according to* $\sigma$*, the number of trees* $I$ *;*

$\sigma$ *←random permutation of [1, n]*

$M_{i} \leftarrow$ *0 for i = 1, …, n*

$$for t \leftarrow1 to I do$$

$for i \leftarrow1 to n do$

$$r_{i}\leftarrow y_{i}-M_{\sigma\left( i \right)-1}\left( X_{i} \right);$$

$$for i \leftarrow1 to n do$$

$\Delta M\leftarrow Learn Model\left[ \left( X_{i}r_{j} \right):\sigma\left( j \right)\leqⅈ \right]$

$M_{i}\to M_{i}+\Delta M$

*Return* $M_{n}$

Please refer to the mentioned paper [[23](#_ENREF_23)] for further information regarding this algorithm and its parameters.

**2.3. XGBoost** [[25](#_ENREF_25)]

The extreme gradient boosting (XGBoost) algorithm, designed and introduced by [Chen and Guestrin [15]](#_ENREF_15), is among the modern machine learning methods based on the gradient boosting decision tree. This algorithm aims to approximate the estimated value to the real value as much as possible by creating a large number of trees (e.g., *k*) in order to minimize errors and maximize adaptability. This algorithm integrates weak learners to create a strong learner. However, weak learners are created through residual fitting in this algorithm [[26](#_ENREF_26),[27](#_ENREF_27)]. XGBoost model extends the cost function of the first-order Taylor and presents the second-order derivative information to make the model converge faster when the model is learning. Due to adding a regularization section to the cost function, the XGBoost algorithm prevents complexity and reduces the risk of overfitting.

The general process of the XGBoost algorithm is as follows:

Regard $D = \left\{ \left( x_{i},y_{i} \right) \right\}$as a dataset with *n* samples and *d* features in each sample. $y_{i}$ represents the target parameter of sample 𝑖. Then, a classification and regression tree (CART) is used as a base model. In the next step, the XGBoost algorithm integrates and mixes *k* base models to estimate the final solution.

$$\hat{y}_{i}=\sum_{k=1}^{K} f_{k}\left( x_{i} \right) (7)$$

where $f_{k}\left( 0 \right)$ is the expression of tree *k* and 𝑘 is the number of trees.

As discussed earlier, regularization is added to the cost function to improve the model performance and reduce its complexity. The regularization section and the cost function will create the XGBoost objective function as mentioned in **Equation (8)**:

$$obj^{\left( k \right)}=\sum_{i=1}^{n} l\left[ y_{i},\hat{y}_{i}^{\left( 1,-1 \right)}+f_{t}\left( x_{i} \right) \right]+\sum_{k} \Omega\left( f_{k} \right) (8)$$

$$\Omega\left( f \right)=\gamma T+\frac{1}{2}\lambda\left\| w \right\|^{2} (9)$$

Supplementary Table S1 introduces all parameters used in the **Equation (8)** and **Equation (9)** briefly.

Supplementary Table S1: list of all parameters used in **Equation (8)** and **Equation (9)**

| sum of the output values of the previous(𝑘−1) trees | $\hat{y}_{i}^{\left( k-1 \right)}$ |
| --- | --- |
| real value | $y_{i}$ |
| predicted value | $\hat{y}_{i}$ |
| output result of tree𝑘 | $f_{k}\left( x_{i} \right)$ |
| differentiable convex loss function | *𝑙* |
| penalty term | $\Omega\left( \cdot\right)$ |
| regularization parameter of leaf weight | *𝛾* |
| regularization parameter of number | *𝜆* |
| Value of the leaf node | *𝑤* |
| Number of the leaf node | *𝑇* |

The objective function after removing the constant term becomes as **Equation (10)**:

$$obj^{\left( k \right)}=\sum_{j=1}^{T} \left[ \left( \Sigma_{i\in I_{j}} g_{i} \right)w_{j}+\frac{1}{2}\left( \Sigma_{i\in I_{j}} h_{i}+\lambda\right)w_{j}^{2} \right]+\gamma T (10)$$

In this equation, $w_{j}$ is the weight of leaf node j. In order to simplify the **Equation (10)**, $H_{i}=\Sigma_{i\in I_{j}} h_{i}{, G}_{i}=\Sigma_{i\in I_{j}} g_{i}$was defined and placed to achieve this equation:

$$obj^{\left( k \right)}=\sum_{j=1}^{T} \left[ {G_{i}w}_{j}+\frac{1}{2}\left( H_{i}+\lambda\right)w_{j}^{2} \right]+\gamma T (11)$$

In **Equation (11)**, the leaf node $w_{j}$ is an uncertain value. Therefore, the objective function $obj^{\left( k \right)}$ is calculated for the first derivative of $w_{j}$, and the optimal value $w_{j}^{*}$ of the leaf node *j* is solved as:

$$w_{j}^{*}=-\frac{G_{i}}{H_{i}+\lambda} (12)$$

To minimize $obj^{\left( k \right)}$, $w_{j}^{*}$ defined as above is now placed in the equation:

$$obj^{\left( k \right)}=-\frac{1}{2}\sum_{j=1}^{T} \frac{G_{i}^{2}}{H_{i}+\lambda}+\gamma T (13)$$

In the aforementioned model, the greedy algorithm is employed to divide the input features when a CART is created. This algorithm is then implemented in the following structure by allocating the parameter *m* to the maximum depth of each tree.

Supplementary Algorithm S3: Exact Greedy Algorithm for Split Finding.

*Input: 𝐼, instance set of the current node*

*Input: 𝑑, feature dimension*

$$gain\leftarrow0$$

$G_{i}$*←* $\Sigma_{i\in I_{j}} g_{i}$ *,* $H_{i}$*←*$\Sigma_{i\in I_{j}} h_{i}$

*for 𝑘 =1 to 𝑚 do*

$G_{L}\leftarrow0,H_{L}\leftarrow0$

*for 𝑗 𝑖𝑛 𝑠𝑜𝑟𝑡𝑒𝑑*$\left( I,byx_{jk} \right)$ *do*

$G_{L}\leftarrow G_{L}+g_{j ,}H_{L}\leftarrow H+h_{j}$

$G_{R}\leftarrow G+G_{L ,}H_{R}\leftarrow H+H_{L}$

$scorⅇ\leftarrow\max\left( score,\frac{G_{L}^{2}}{H_{L}+\lambda}+\frac{G_{R}^{2}}{H_{R}+\lambda}-\frac{G^{2}}{H+\lambda} \right)$

*end*

*end*

*Output: Split with max score*

In every iteration, the above algorithm navigates the features of each node from the root node. Then, for choosing the split node we use the point that has the highest score. Splitting continues to the maximum depth of the tree and then building the residue of the next tree starts. Finally, the XGBoost model is developed by collecting and using the resultant trees simultaneously. Supplementary Figure S2. demonstrates the proposed algorithm structure from a different perspective for a simpler and more tangible understanding [[28](#_ENREF_28)].





Supplementary Figure S2. schematic of XGBoost algorithm

# **3. Comparison with the preexisting approaches**

After developing the models and finding that XGBoost is the best paradigm, we have compared this algorithm with various available approaches for predicting the formation volume factor (B_o_). Supplementary Table S2 and Supplementary Table S3 show the superiority of the XGBoost model in comparison with previous machine learning methods and various equations of state using the statistical indicators.

Supplementary Table S2: Summary of Previous Attempts toward Estimating B_o_ Using ML.

| **Author** | **Algorithm** | **Error (AARD %)** |
| --- | --- | --- |
| [BC Gharbi, et al. [29]](#_ENREF_29) | ANNs | 2.79 |
| [Elsharkawy [30]](#_ENREF_30) | RBF | 0.53 |
| [Mahdiani, et al. [31]](#_ENREF_31) | SA | 1.25 |
| [Fattah, et al. [32]](#_ENREF_32) | GP | 0.33 |
| [Elkatatny, et al. [33]](#_ENREF_33) | ANNs | 0.99 |
| [Saghafi, et al. [34]](#_ENREF_34) | ANFIS | 1.80 |
| [Seyyedattar, et al. [35]](#_ENREF_35) | ET | 0.099 |
| [Rashidi, et al. [36]](#_ENREF_36) | PSO-MELM | Not reported |
| [Tariq, et al. [37]](#_ENREF_37) | PSO-MELM | Not reported |
| [Larestani, et al. [38]](#_ENREF_38) | Lumped-ETs | 1.168 |
| This Study | XGBoost | 0.259 |

Supplementary Table S3: Statistical Evaluation of Equations of States in Estimating B_o_.

| **Method** | **AARD (%)** | **RMSE** | **R^2^** |
| --- | --- | --- | --- |
| Redlich-Kwong | 8.7154 | 0.3254 | 0.7894 |
| 2-parameter Peng-Robinson | 8.5179 | 0.1824 | 0.7452 |
| 2-parameter Soave-Redlich-Kwong | 7.9151 | 0.1708 | 0.7767 |
| 3-parameter Soave-Redlich-Kwong | 5.2804 | 0.1426 | 0.8444 |
| Schmidt-Wenzel | 4.9487 | 0.1468 | 0.8350 |
| Zudkevitch-Joffe | 4.8136 | 0.1590 | 0.8064 |
| 3-parameter Peng-Robinson | 4.2345 | 0.1095 | 0.9083 |

**References**

1 Schapire, R. E. The boosting approach to machine learning: An overview. *Nonlinear estimation and classification*, 149-171 (2003).

2 Syarif, I., Zaluska, E., Prugel-Bennett, A. & Wills, G. in *Machine Learning and Data Mining in Pattern Recognition: 8th International Conference, MLDM 2012, Berlin, Germany, July 13-20, 2012. Proceedings 8.* 593-602 (Springer).

3 Lee, K. C. & Cho, H. Performance of ensemble classifier for location prediction task: emphasis on Markov Blanket perspective. *International Journal of u-and e-Service, Science and Technology* **3**, 2010 (2010).

4 Polikar, R. Ensemble based systems in decision making. *IEEE Circuits and systems magazine* **6**, 21-45 (2006).

5 Dietterich, T. G. in *International workshop on multiple classifier systems.* 1-15 (Springer).

6 Gudadhe, M., Prasad, P. & Wankhade, L. K. in *2010 International Conference on Computer and Communication Technology (ICCCT).* 731-735 (IEEE).

7 Cup, K. <http://kdd>.ics.uci.edu/databases/kddcup99/kddcup99. html. *The UCI KDD Archive* (1999).

8 Tavallaee, M., Bagheri, E., Lu, W. & Ghorbani, A. A. in *2009 IEEE symposium on computational intelligence for security and defense applications.* 1-6 (Ieee).

9 Breiman, L. Bagging predictors. *Machine learning* **24**, 123-140 (1996).

10 Zhou, Z.-H. Encyclopedia of Biometrics. *Ensemble Learning* (2009).

11 Breiman, L. Random forests. *Machine learning* **45**, 5-32 (2001).

12 Geurts, P., Ernst, D. & Wehenkel, L. Extremely randomized trees. *Machine learning* **63**, 3-42 (2006).

13 Bartlett, P., Freund, Y., Lee, W. S. & Schapire, R. E. Boosting the margin: A new explanation for the effectiveness of voting methods. *The annals of statistics* **26**, 1651-1686 (1998).

14 Graczyk, M., Lasota, T., Trawiński, B. & Trawiński, K. in *Intelligent Information and Database Systems: Second International Conference, ACIIDS, Hue City, Vietnam, March 24-26, 2010. Proceedings, Part II 2.* 340-350 (Springer).

15 Chen, T. & Guestrin, C. in *Proceedings of the 22nd acm sigkdd international conference on knowledge discovery and data mining.* 785-794.

16 Karthikeyan, A. & Priyakumar, U. D. Artificial intelligence: machine learning for chemical sciences. *Journal of Chemical Sciences* **134**, 1-20 (2022).

17 Syarif, I., Zaluska, E., Prugel-Bennett, A. & Wills, G. in *International Workshop on Machine Learning and Data Mining in Pattern Recognition.* 593-602 (Springer).

18 Al Daoud, E. Comparison between XGBoost, LightGBM and CatBoost using a home credit dataset. *International Journal of Computer and Information Engineering* **13**, 6-10 (2019).

19 Habib, A.-Z. S. B., Tasnim, T. & Billah, M. M. in *2019 2nd International Conference on Innovation in Engineering and Technology (ICIET).* 1-6 (IEEE).

20 Nie, P., Roccotelli, M., Fanti, M. P., Ming, Z. & Li, Z. Prediction of home energy consumption based on gradient boosting regression tree. *Energy Reports* **7**, 1246-1255 (2021).

21 Friedman, J. H. Greedy function approximation: a gradient boosting machine. *Annals of statistics*, 1189-1232 (2001).

22 Dorogush, A. V., Ershov, V. & Gulin, A. CatBoost: gradient boosting with categorical features support. *arXiv preprint arXiv:1810.11363* (2018).

23 Huang, G. *et al.* Evaluation of CatBoost method for prediction of reference evapotranspiration in humid regions. *Journal of Hydrology* **574**, 1029-1041 (2019).

24 Prokhorenkova, L., Gusev, G., Vorobev, A., Dorogush, A. V. & Gulin, A. CatBoost: unbiased boosting with categorical features. *Advances in neural information processing systems* **31** (2018).

25 Liu, Y. *et al.* Research on the prediction of green plum acidity based on improved XGBoost. *Sensors* **21**, 930 (2021).

26 Xiao, Z. & Luo, A. l. XGBoost based stellar spectral classification and quantized feature. *Spectroscopy and Spectral Analysis* **39**, 3292-3296 (2019).

27 Zopluoglu, C. Detecting examinees with item preknowledge in large-scale testing using extreme gradient boosting (XGBoost). *Educational and psychological measurement* **79**, 931-961 (2019).

28 Mo, H., Sun, H., Liu, J. & Wei, S. Developing window behavior models for residential buildings using XGBoost algorithm. *Energy and Buildings* **205**, 109564 (2019).

29 BC Gharbi, R. & Elsharkawy, A. M. Neural network model for estimating the PVT properties of Middle East crude oils. *SPE Reservoir Evaluation & Engineering* **2**, 255-265 (1999).

30 Elsharkawy, A. M. in *SPE Asia Pacific oil and gas conference and exhibition.* SPE-49961-MS (SPE).

31 Mahdiani, M. R. & Norouzi, M. A new heuristic model for estimating the oil formation volume factor. *Petroleum* **4**, 300-308 (2018).

32 Fattah, K. & Lashin, A. Improved oil formation volume factor (Bo) correlation for volatile oil reservoirs: An integrated non-linear regression and genetic programming approach. *Journal of King Saud University-Engineering Sciences* **30**, 398-404 (2018).

33 Elkatatny, S. & Mahmoud, M. Development of new correlations for the oil formation volume factor in oil reservoirs using artificial intelligent white box technique. *Petroleum* **4**, 178-186 (2018).

34 Saghafi, H. R., Rostami, A. & Arabloo, M. Evolving new strategies to estimate reservoir oil formation volume factor: Smart modeling and correlation development. *Journal of Petroleum Science and Engineering* **181**, 106180 (2019).

35 Seyyedattar, M., Ghiasi, M. M., Zendehboudi, S. & Butt, S. Determination of bubble point pressure and oil formation volume factor: Extra trees compared with LSSVM-CSA hybrid and ANFIS models. *Fuel* **269**, 116834 (2020).

36 Rashidi, S. *et al.* Determination of bubble point pressure & oil formation volume factor of crude oils applying multiple hidden layers extreme learning machine algorithms. *Journal of Petroleum Science and Engineering* **202**, 108425 (2021).

37 Tariq, Z., Mahmoud, M. & Abdulraheem, A. Machine learning-based improved pressure–volume–temperature correlations for black oil reservoirs. *Journal of Energy Resources Technology* **143**, 113003 (2021).

38 Larestani, A., Hemmati-Sarapardeh, A., Samari, Z. & Ostadhassan, M. Compositional Modeling of the Oil Formation Volume Factor of Crude Oil Systems: Application of Intelligent Models and Equations of State. *ACS omega* **7**, 24256-24273 (2022).
